# An inducible CRISPRi system for phenotypic analysis of essential genes in *Pseudomonas aeruginosa*

**DOI:** 10.1128/mbio.02767-25

**Published:** 2026-02-11

**Authors:** Jaryd R. Sullivan, Kristina M. Ferrara, Rebecca Barrick, Keith P. Romano, Thulasi Warrier, Deborah T. Hung

**Affiliations:** 1 Department of Molecular Biology, Center for Computational and Integrative Biology, Massachusetts General Hospital 2348, Boston, Massachusetts, USA; 2 The Broad Institute of MIT and Harvard, Cambridge, Massachusetts, USA; 3 Department of Genetics, Harvard Medical School 1811 , Boston, Massachusetts, USA; 4 Division of Pulmonary and Critical Care Medicine, Brigham and Women’s Hospital, Boston, Massachusetts, USA; NYU Langone Health, New York, New York, USA

**Keywords:** CRISPRi, genetic perturbation, transcriptomics, *Pseudomonas aeruginosa*

## Abstract

Precise and tunable genetic tools are essential for high-throughput functional genomics. To address this need in the important gram-negative pathogen *Pseudomonas aeruginosa*, we developed and characterized a tightly regulated CRISPR-interference (CRISPRi) system that enables precise and tunable repression of essential genes. The system utilizes a rhamnose-inducible promoter to control both the *Streptococcus pasteurianus*-derived dCas9 and gene-specific sgRNAs, each encoded on separate plasmids for modularity and efficiency. The combination of tight regulation and high conjugation efficiency facilitated the rapid and facile construction of strains with regulated depletion of 16 essential genes spanning diverse pathways. Comparison of phenotypes across the different genetically depleted strains, including growth rate, susceptibility to antibiotics, and changes in transcriptional programs, revealed novel aspects of gene function or small-molecule mechanism of action. Finally, the rhamnose-inducible CRISPRi system supports the generation and stable maintenance of pooled mutant libraries, thereby paving the way for future genome-wide, systematic assessment of individual gene vulnerabilities, which will provide critical insights for target prioritization in antibiotic discovery efforts against this recalcitrant pathogen.

**IMPORTANCE:**

CRISPR-interference (CRISPRi) has become an invaluable tool for studying genetics. In particular, the ability to knockdown (KD) genes enables the study of essential genes and their role in cell survival. However, a tightly regulated gene KD system is required to gain valuable insights into these vulnerable genes by virtue of their essentiality. We report a tightly regulated CRISPRi system to study the biology of essential gene perturbations in *Pseudomonas aeruginosa*, an important gram-negative pathogen that causes severe infections and is increasingly resistant to current antibiotics. This system enables characterization of both chemical genetic interactions between small molecules and specific gene depletions and the impact of genetic perturbations on transcriptional networks. Genetic perturbations using CRISPRi can thus further our understanding of basic biology with translation toward future antimicrobial development.

## INTRODUCTION

Bacterial genetics has a long history of enabling the elucidation of individual gene roles in critical cellular functions and complex phenotypes that shape microbial physiology, pathogenesis, and antibiotic resistance. In the past, genetic strategies for gene disruption have included targeted allelic exchange with two-step homologous recombination, phage recombinase-based single-step replacement with marker genes, as well as nontargeted approaches using transposon insertion mutagenesis for gene disruption (1–3). Although well-suited for studying non-essential genes that are dispensable for bacterial growth, these approaches are more cumbersome when applied to essential genes where graded depletion rather than total disruption is often desired. Genetic strategies for essential gene depletion have historically required replacing the native promoter with an inducible promoter or inserting protein degradation tags to control the level of expression (4–6). Recently, however, the advent of CRISPR-interference (CRISPRi) technology has transformed the ability to perturb essential genes in a scalable fashion (7, 8). By programming a catalytically inactive Cas9 (dCas9) to bind—but not cut—specific DNA sequences guided by an sgRNA, transcription of the target gene can be silenced through steric hindrance that blocks RNA polymerase at the site.

CRISPRi has proven to be a valuable resource in the field of bacterial genetics with well-characterized systems developed for model organisms like *Escherichia coli* (7, 9, 10), *Bacillus subtilis* (11), and *Streptococcus pneumoniae* (12) with the power to study the biological functions of essential genes and perform CRISPRi screens. A mobile CRISPRi system was created to further facilitate rapid CRISPRi implementation into a variety of other gram-positive and negative bacteria with an inducible *dcas9* from *Streptococcus pyogenes* and constitutively expressed sgRNA (13). While this *S. pyogenes* dCas9-based system was less effective in *P. aeruginosa* due to observed instability of the dCas9 protein (13), a more optimized *S. pyogenes* system was recently reported (14). A different CRISPRi tool based on *Streptococcus pasteurianus* dCas9 has also been reported in *Pseudomonas* spp. with up to 1,000-fold reduction in targeted activity, but the expression of *dcas9* from anhydrotetracycline or IPTG-inducible promoters was leaky, which makes targeting essential genes more challenging (15). Activation of the system in the absence of an inducer can preclude the construction, isolation, and thus study of depletion mutants corresponding to essential genes due to uncontrolled target depletion, leading to a constitutive fitness cost. This fitness cost can result in selection for suppressor mutations just from simple propagation of the hypomorphic strain; can preclude the shotgun generation of comprehensive, pooled, genome-wide libraries targeting essential genes as less fit mutants will be competed out by more fit ones; or can preclude construction of the strain altogether. In *Mycobacteria* spp., this problem was solved by tightly controlling both *dcas9* from *Streptococcus thermophilus* and sgRNA expression from the engineered anhydrotetracycline-driven promoters (16). This advancement enabled the successful evaluation not simply of gene essentiality but also the relative vulnerability of the essential genes on a genome-wide scale (17). A similar strategy with an engineered tetracycline-inducible promoter aided optimization of the *S. pyogenes* dCas9-based tool in *P. aeruginosa* (14).

Here, we developed a tightly regulated CRISPRi system with the *S. pasteurianus* dCas9 to enable phenotypic profiling of individual essential genes or pooled CRISPRi studies in *P. aeruginosa*, a WHO-prioritized pathogen for urgent antimicrobial discovery (18). It is based on a two-piece modular strategy with dual inducible-promoter control of the catalytically inactive dCas9 and the sgRNA. Unlike many of the CRISPRi systems that rely on induction of *dcas9* and constitutive expression of the sgRNA, we utilized the rhamnose-inducible P_rhaBAD_ promoter for both *dcas9* and sgRNA expression (19–21). This system provided tight control with increased knockdown (KD) efficiency in the presence of inducer and minimal KD in the absence of an inducer, thus facilitating detailed functional characterization of essential genes. In addition, we separated *dcas9* from the sgRNA, which enabled the generation of a parental *P. aeruginosa::dcas9* strain into which smaller sgRNA-carrying plasmids could be integrated with greater success, thereby enabling construction of pooled CRISPRi libraries targeting essential genes rapidly and with ease. Finally, we demonstrated the tunability of this tool both by partially depleting antibiotic targets to explore chemical genetic interactions with anti-pseudomonal drugs and by maximally depleting essential genes to identify perturbation-specific transcriptional responses in *P. aeruginosa*.

## RESULTS

### Assessing different dCas9 for optimizing an inducible CRISPRi system in *Pseudomonas aeruginosa*

**Fig 1:**
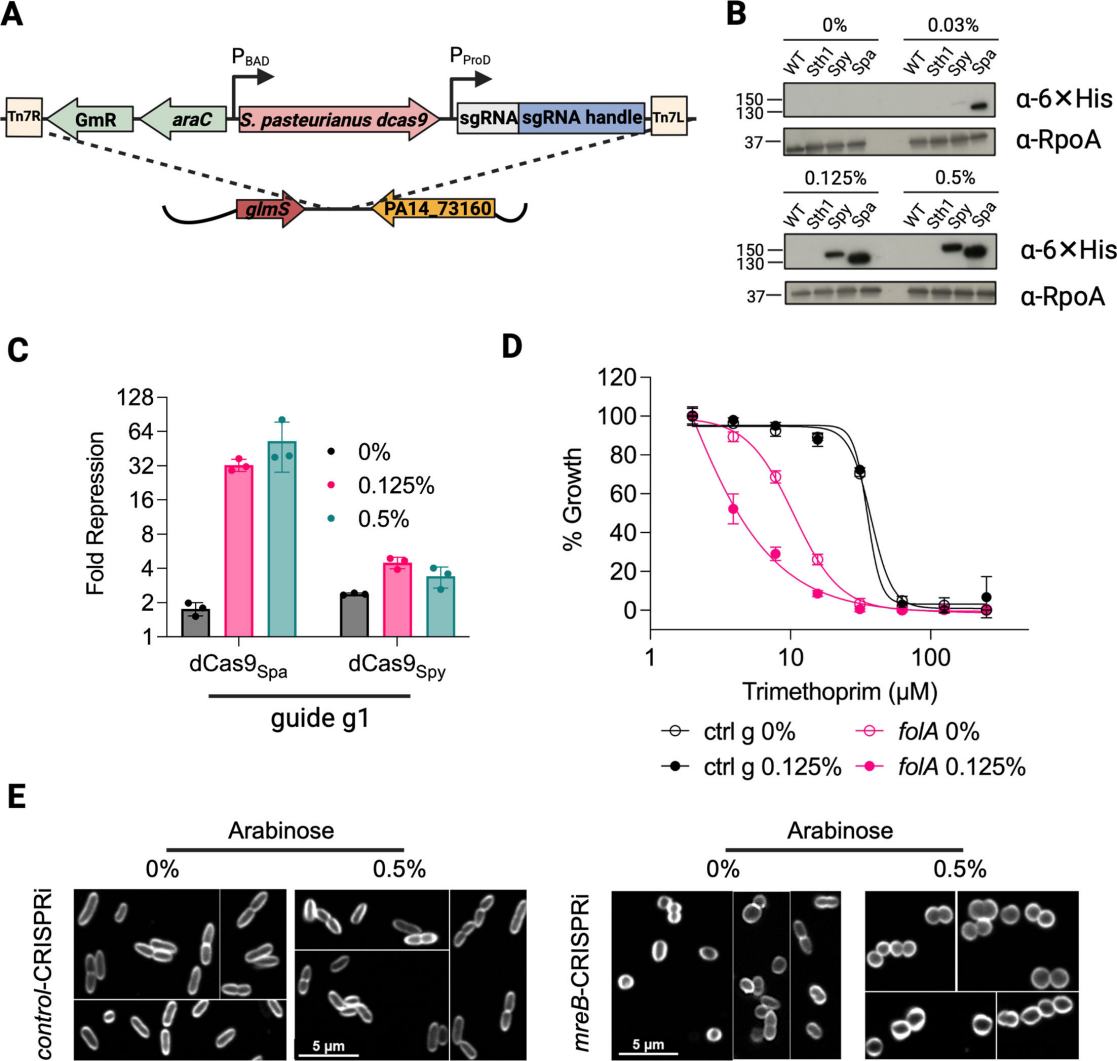
An arabinose-based CRISPRi system yields leaky CRISPRi phenotypes. (**A**) Schematic of the CRISPRi P_araBAD_ system (CRISPRi^A^). dCas9 expression is driven by the arabinose-inducible promoter P_araBAD,_ with sgRNA constitutively expressed under the P_ProD_ promoter. The CRISPRi^A^ system integrates at the attTn7 site in the PA14 genome. (**B**) Western blot analysis of the expression of C-terminal 6×His-tagged dCas9 variants in the PA14 strain at varying arabinose concentrations. Wild-type PA14 or PA14 expressing dCas9 from *S. thermophilus* (Sth1), *S. pyogenes* (Spy), or *S. pasteurianus* (Spa) was lysed after induction with 0, 0.03, 0.125, or 0.5% arabinose and blotted with an anti-His antibody. dCas9_Spa_ and dCas9_Spy_ levels were arabinose dose-dependent with a higher abundance of dCas9_Spa_ while dCas9_Sth1_ was not expressed. (**C**) mKate2 fluorescence repression (normalized to control sgRNA) by CRISPRi^A^ using dCas9_Spa_ or dCas9_Spy_ with sgRNA g1 (Fig. S1A) measured after 15 h of growth in M9 minimal media with 0, 0.125, or 0.5% arabinose. dCas9_Spa_ induction led to stronger depletion of mKate2 than dCas9_Spy_, whose activity was also not arabinose-dependent. (**D**) Sensitization of *folA*-CRISPRi^A^ to trimethoprim. Activity of trimethoprim against CRISPRi^A^ strains with control or *folA*1 (Fig. S1H) sgRNAs grown in lysogeny broth (LB) with and without 0.125% arabinose for 24 h. Trimethoprim sensitization was dependent on having the *folA*1 guide but independent of arabinose. (**E**) Morphology of CRISPRi^A^ strains with control or *mreB* sgRNAs. CRISPRi^A^ strains were grown in 0% or 0.5% arabinose for ~19h, stained with FM-143x, and visualized using confocal microscopy. *mreB*-CRISPRi^A^ cells displayed spherical morphology even in the absence of arabinose.

To develop a tightly regulated CRISPRi system for essential gene perturbations in *P. aeruginosa*, we chose to focus on systems with inducers that are minimally impacted by efflux pumps in *P. aeruginosa* to preserve their utility in a wide diversity of strains including clinical isolates that often display hyper-efflux phenotypes (22, 23). We started with the arabinose-inducible P_araBAD_ promoter to drive *dcas9* since the IPTG-inducible P_lac_/P_tac_ promoters had been reported to cause leaky CRISPRi activation in *P. aeruginosa* (15). We paired arabinose-inducible *dcas9* expression with a constitutive P_ProD_ promoter driving sgRNA expression (termed CRISPRi^A^, for arabinose). Both were incorporated into a single *att*Tn7 site-integrating mini-Tn7 vector (24) and introduced into *P. aeruginosa* strain PA14 (Fig. 1A). When we compared codon-optimized, His-tagged *dcas9* variants shown to have varying degrees of activity in different species of bacteria (15, 16, 25, 26), including dCas9 from *S. pasteurianus* (dCas9_Spa_), *S. pyogenes* (dCas9_Spy_), and *S. thermophilus* (dCas9_Sth1_), we found by Western blot analysis using an anti-His antibody that dCas9_Spa_ showed the strongest arabinose dose-dependent expression levels, followed by dCas9_Spy_, while there were no detectable levels of dCas9_Sth1_ (Fig. 1B ; Fig. S1).

To compare gene KD efficiency, we generated a reporter system based on the fluorescent protein mKate2 (27, 28) and designed guides to the promoter and along the length of the *mkate2* coding sequence (Fig. S2A). Not only did dCas9_Spa_ repress mKate2 fluorescence more than dCas9_Spy_ (~50- vs 4-fold), but it showed less than a 2-fold repression in the absence of the inducer and greater inducer dose-dependence than the *S. pyogenes*-based system, thus demonstrating tighter control (Fig. 1C; Fig. S2B). Western blot analysis revealed that dCas9_Spy_ likely undergoes some degree of degradation in *P. aeruginosa,* as evidenced at higher arabinose concentrations (Fig. S1 and S2C) (13). We therefore focused on the dCas9_Spa_-based system for further characterization and optimization, consistent with a previous report using dCas9_Spa_ (15).

### Expanding the functional PAM variants for dCas9_Spa_ in *P. aeruginosa*

Many factors, including proto-adjacent spacer motif (PAM) sequence, guide sequence location within the gene, guide length, and template versus nontemplate strand targeting, influence CRISPRi efficiency (7). Three dCas9_Spa_-specific PAM sequences have been previously reported (15). To try to expand the number of PAM sites that could be targeted in the *P. aeruginosa* genome, we systematically explored variations in the PAM sequence using the mKate2 reporter assay. We tested the three previously reported dCas9_Spa_-specific PAM sequences (15) (NNGTGA, NNGCGA, and NNGTAA) and seven additional variants to expand the range of the guide sequence and location. Previous sequences NNGTGA and NNGCGA indeed provided the highest degrees of fluorescence repression (~8- to 64-fold) irrespective of the guide location (Fig. S2D). On the other hand, while NNGTAA generally exhibited lower repression (~2- to 8-fold), its activity was location-dependent, with the guide located 48 bp into the open reading frame of mKate2 (g5) providing the strongest repression of all tested guides (Fig. S2D). We identified a new PAM, NNGTAT, with efficiency identical to the known NNGTAA, thus expanding the repertoire of dCas9_Spa_-specific PAMs and the number of PAM sites in the *P. aeruginosa* genome by an additional 10% (Fig. S2E). As expected, changing the first specific nucleotide or more than one nucleotide in these PAM sequences, when introduced in the *mKate2* transcription start site, completely abrogated dCas9 activity; similarly, targeting the template strand resulted in no activity, as evidenced by the lack of activity of a guide designed to do so (sgRNA g12; Fig. S2D) (16, 29). Notably, variability in the length of the specificity region of the sgRNA from the standard 21–24 nucleotide (nt) to 16 nt did not alter KD efficiency (Fig. S2F). Based on these findings, we prioritized the three known PAMs during guide design to select 20–24-nt-long guides to target the nontemplate strand of genes of interest at sites most proximal to the transcriptional start site. Although we defined a new PAM, NNGTAT, it was not included due to its reduced strength.

### Activity of the arabinose-dependent dCas9_Spa_ system occurred in the absence of inducer

Next, we evaluated the system’s efficacy toward endogenous essential genes in the PA14 strain. Targeting the dihydrofolate reductase gene, *folA*, with three sgRNAs that bound different locations led to variable growth defects compared to the *control*-CRISPRi^A^ strain (Fig. S2G). While in the absence of arabinose, all three strains grew normally, in the presence of arabinose, guide 1 located in the promoter region led to the strongest growth deficit, guide 2 located 28 bp into the open reading frame had a milder impact, and guide 3 located 159 bp into the open reading frame had no effect (Fig. S2H). However, while normal strain growth in the absence of the inducer was encouraging, in a different, more sensitive assay in which we measured the MIC_90_ of a FolA inhibitor (trimethoprim) in the CRISPRi^A^ mutants, we observed lower MIC_90_ values in the *folA*-CRISPRi^A^ strain relative to the *control*-CRISPRi^A^ strain, even in the absence of the inducer (Fig. 1D). This increased trimethoprim sensitivity implied that there was some baseline level of FolA depletion despite the apparent normal growth, indicating leaky activation of the CRISPRi^A^ system. The observed leakiness was not just isolated to *folA*. We similarly observed phenotypic consequences in the absence of the inducer in a strain designed to deplete *mreB*, which controls the rod shape of gram-negative bacteria. In uninduced conditions, *mreB*-CRISPRi^A^ bacteria also had normal growth based on the optical density (OD_600_); however, they had a spherical morphology, in contrast to the typical rod shape of wild-type bacteria and the control-CRISPRi^A^ strain, suggesting some degree of target depletion (Fig. 1E).

### Development of a stronger and tighter CRISPRi system in *P. aeruginosa*

**Fig 2:**
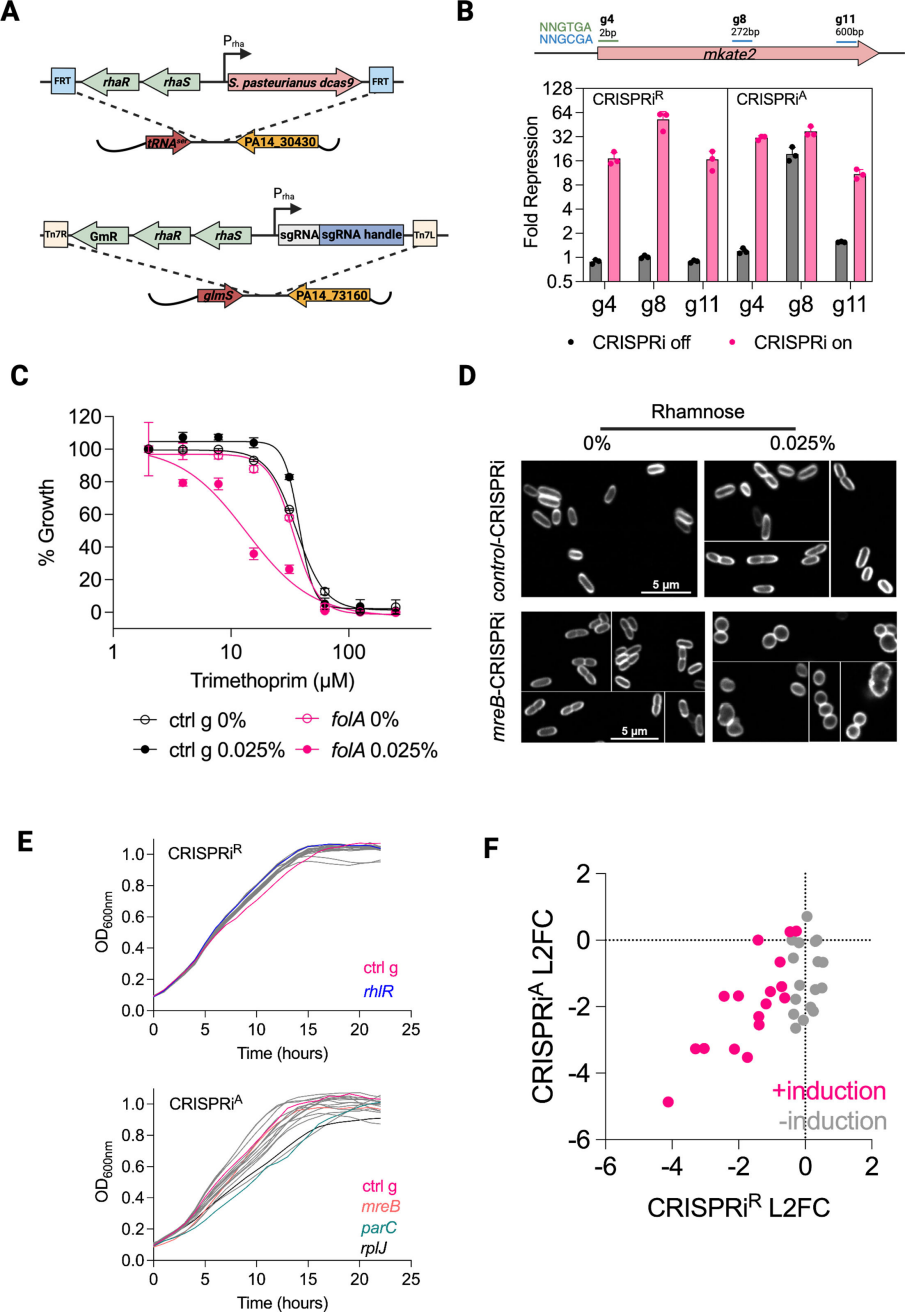
A rhamnose-based, modular two-piece CRISPRi P_rhaBAD_ system (CRISPRi^R^) eliminates leaky phenotypes. (**A**) Schematic of the CRISPRi^R^ system. dCas9_Spa_ expression is driven by the rhamnose-inducible promoter P_rhaBAD_ and is integrated at the ΦCTX site in the PA14 genome. This establishes a parental dCas9_Spa_ expressing strain of PA14. sgRNAs are also under control of the P_rhaBAD_ promoter and are integrated at the attTn7 site in the PA14 genome. (**B**) In the presence of the inducer, CRISPRi^R^ activity against the mKate2 reporter is comparable to that of CRISPRi^A^, but in the absence of the inducer, CRISPRi^R^ is more tightly off. mKate2 fluorescence repression (normalized to control sgRNA) by CRISPRi with sgRNAs g4, g8, and g11 measured after 15 h of growth in minimal M9 media with 0.05% rhamnose (CRISPRi^R^) or 0.5% arabinose (CRISPRi^A^). (**C**) Sensitization of *folA*-CRISPRi^R^ to trimethoprim. Activity of trimethoprim against CRISPRi^R^ strains with control or *folA*1 sgRNAs (Fig. S1H) grown in LB for 24 h. Trimethoprim sensitization was dependent on expressing the *folA*1 sgRNA and on the presence of rhamnose. (**D**) Morphology of CRISPRi^R^ strains with control or mreB sgRNAs. CRISPRi^R^ strains were grown in 0% or 0.05% rhamnose for ~19 h, stained with FM-143x, and visualized using confocal microscopy. mreB-CRISPRi^R^ cells displayed spherical morphology only in the presence of the CRISPRi^R^ inducer, indicating a tight off state. (**E**) Comparison of CRISPRi growth curves in LB without the inducer. CRISPRi^R^ strains targeting essential genes exhibited uniform growth in the absence of rhamnose, while CRISPRi^A^ strains exhibited variable growth in the absence of arabinose. Each line is the growth curve for a different CRISPRi strain with the control-CRISPRi strain shown in pink. (**F**) Increased control of the CRISPRi^R^ system limits leaky KD of target genes. CRISPRi KD of target genes analyzed by RNA-seq of each corresponding CRISPRi depletion strain. The degree of KD of the targeted gene is quantified using log_2_ fold change (L2FC) relative to control-CRISPRi strain after 6 h of growth in 0.5% arabinose (CRISPRi^A^) or 0.05% rhamnose (CRISPRi^R^). Each dot represents one CRISPRi strain either in induced (pink) or uninduced conditions (gray). Without induction, there is no decrease in the expression of the CRISPRi^R^ targeted gene in the corresponding strain, in contrast to the CRISPRi^A^ system.

To create a more tightly regulated system, we substituted the arabinose P_araBAD_ promoter with the rhamnose-inducible P_rhaBAD_ promoter, which is known to be more tightly regulated in *P. aeruginosa* (19, 30). Additionally, we used the P_rhaBAD_ promoter to regulate the expressions of both *dcas9* and the guide. We designed it as a two-plasmid system to make it more modular and avoid cloning inefficiency due to the large size of the mini-Tn7 vector when the P_rhaBAD_ promoter elements were introduced. The *dcas9*_Spa_ was integrated at the ϕ*CTX* attachment site in the wild-type PA14 genome, thereby generating a parental CRISPRi strain (PA14::*dcas9*). Different sgRNAs were then separately and easily integrated at the *att*Tn7 site using a smaller mini-Tn7 plasmid (Fig. 2A). KD efficiency of this P_rhaBAD_-based system (termed CRISPRi^R^, for rhamnose) was comparable to the P_araBAD_ system on mKate2 repression (Fig. 2B); however, activation of the system was now tightly dependent on rhamnose, as evidenced by the strict rhamnose-dependence of trimethoprim sensitization of *folA*-CRISPRi^R^ and the absence of morphological changes observed in *mreB*-CRISPRi^R^ strains in the absence of rhamnose (Fig. 2C and D).

### Targeting an expanded set of endogenous genes in *P. aeruginosa*

**Fig 3:**
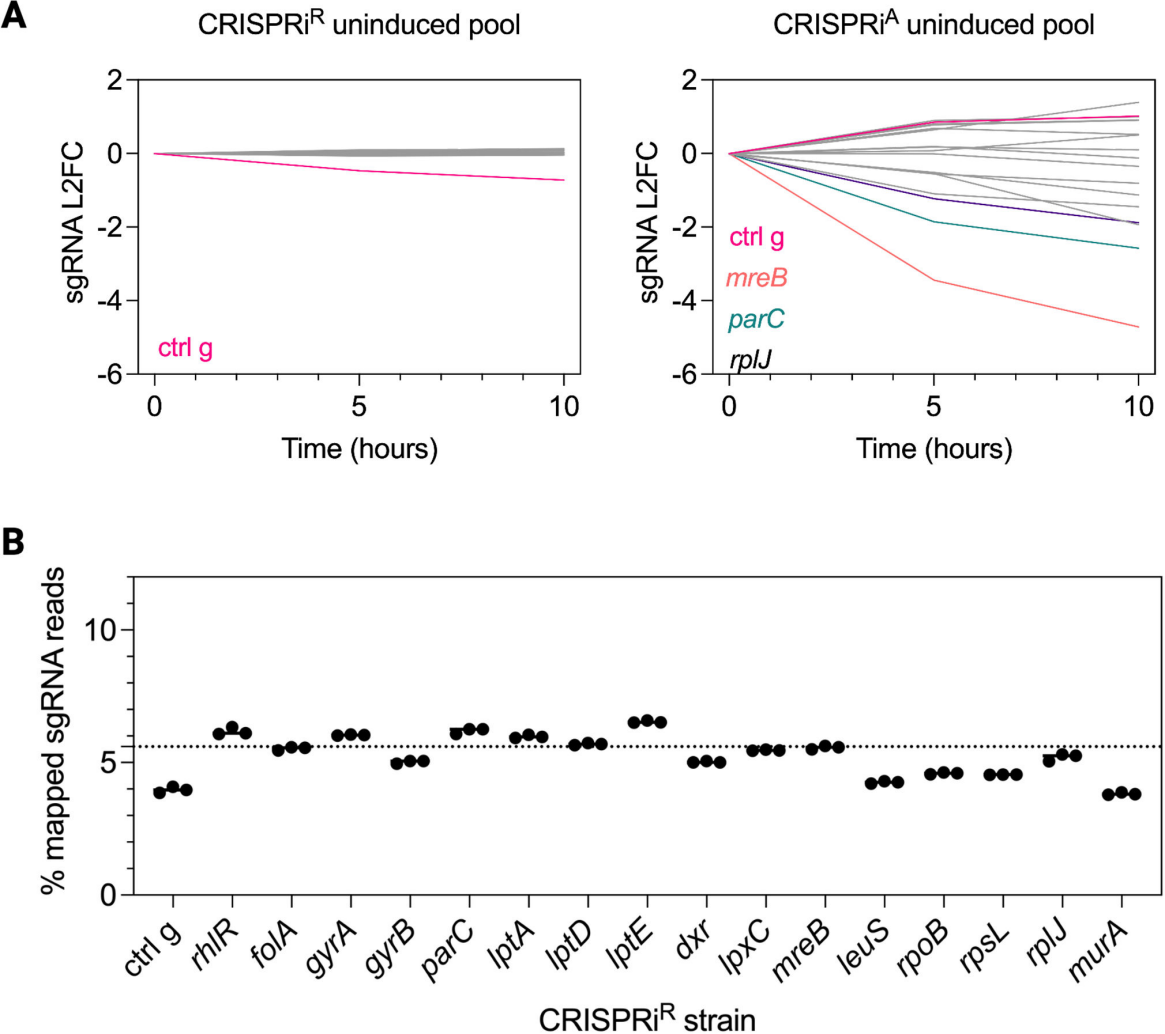
Tight control of CRISPRi^R^ facilitates pooled CRISPRi library construction and propagation. (**A**) Targeted amplicon sequencing of sgRNAs as a proxy for individual strain census within a propagated pool of CRISPRi^R^ or CRISPRi^A^ strains without the inducer. Eighteen individual CRISPRi strains were pooled at equal ratios at time zero. No loss of strains was detected in the CRISPRi^R^ pool even after 10 h of growth, as determined by L2FC in the sgRNA amplicon abundance corresponding to the engineered strain targeting each essential gene (left). In contrast, strain dropout was apparent in the pool of CRISPRi^A^ strains (right). Each line represents a different CRISPR mutant, with colored lines referring to the corresponding colored gene shown. (**B**) Pooled construction of a single CRISPRi^R^ library containing 17 different strains. Conjugation was carried out with a pool of 17 different *E. coli* sgRNA donor strains (pooled at equal ratios). Targeted amplicon sequencing to measure the abundance of sgRNAs as a proxy for strain abundance is shown as a percentage of all mapped sgRNA reads (dotted line indicates 5.8% theoretical target for each CRISPRi^R^ sgRNA). All the individual strains were recovered at the expected ~5% of the total pool abundance.

We compared the new rhamnose-based system with the arabinose system by individually constructing, in parallel, 16 genes that are part of the essential core genome of *P. aeruginosa* (31). Selected genes are involved in DNA synthesis (*folA*, *gyrA*, *gyrB*, and *parC*), protein synthesis (*rpoB*, *rpsL*, *rplJ*, and *leuS*), and cell envelope biogenesis (*lptA*, *lptD*, *lptE*, *dxr*, *lpxC*, *mreB*, *murA*, and *pbpA*). We included the non-essential transcription factor *rhlR*, which coordinates pyocyanin production as a negative control, with KD resulting in visible reduction in cyan-pigmented pyocyanin in the medium in a rhamnose dose-dependent manner (Fig. S3A). The tightness of the new CRISPRi^R^ system was validated by observing more uniform growth curves across the 18 CRISPRi strains in the “off” state compared to the leakier CRISPRi^A^ system (Fig. 2E). Enumeration of transcript levels of each target gene by RNA-seq showed that there was no gene depletion in the corresponding CRISPRi strain using the CRISPRi^R^ system in the absence of rhamnose, in contrast to measurable gene depletion in the corresponding strain using the CRISPRi^A^ system in the absence of arabinose (Fig. 2F). We also compared the regulation of the arabinose and rhamnose CRISPRi systems by propagating the 18 CRISPRi strains uninduced in a pooled fashion. CRISPRi strains were grown separately without the inducer and pooled at equal density and continuously passaged without the inducer for up to 10 h (Fig. S4A). We sampled the pools over time, amplified the sgRNAs from the pool, and sequenced the amplicon pools to enumerate the surviving census of each strain in the pool. We observed dropout of certain strains over time using the CRISPRi^A^ system, which confirmed reduced fitness of some strains due to leaky downregulation of their corresponding targets even in the absence of the inducer (Fig. 3A). On the other hand, pool diversity with equal representation of each strain was maintained with the CRISPRi^R^ system (Fig. 3A).

To further support the benefit of a tightly regulated system, we were able to not simply propagate but also directly generate in a single pool a library of CRISPRi^R^ strains targeting these essential genes (Fig. S4B). First, we cloned sgRNAs individually into the miniTn7 sgRNA vector in the *E. coli* donor strain. Second, we set up a tetrapartite mating strategy with a recipient PA14::*dcas9* strain; two *E. coli* helper strains harboring the helper plasmids pRK2013 and pTNS2, respectively; and a pool of *E. coli* donor strains mixed at equal proportions carrying the CRISPRi^R^ sgRNA vectors. After selecting for successful PA14 conjugants on irgasan and gentamicin, all colonies were pooled together for targeted amplicon sequencing of the guides. With 17 different sgRNAs in the pool, we expected each sgRNA to make up 5.8% of all total mapped reads (dashed gray line), and indeed, we obtained ~5% mapped sgRNA reads for each sgRNA (Fig. 3B). The tightly regulated rhamnose system thus not only minimized loss of CRISPRi^R^ strain diversity with propagation over time but enabled construction of a strain pool with equal representation of individual CRISPRi^R^ strains.

### Growth phenotypes of essential genes depleted in *P. aeruginosa*

Upon activation of the CRISPRi^R^ machinery, we observed a range of gene KD levels and growth phenotypes across the different gene targets, despite selecting optimal PAMs that were most proximal to the transcription/translation start site and using one of the three strongest PAMs for all genes (Fig. S3B). The relationship between gene identity, degree of KD, and impact on phenotypes including growth is complicated, captured in the concept of gene vulnerability and the sensitivity of the cell to target depletion (17, 32). We observed variable growth phenotypes even when knocking down targets involved in the same pathway or function.

We observed that gene depletion can lead to distinct phenotypic outcomes, ranging from bactericidal effects to bacteriostatic behavior. When we knocked down *murA*, which is involved in peptidoglycan synthesis, we observed growth arrest in contrast to the bactericidal phenotype resulting from depletion of *lpxC*, which is involved in lipopolysaccharide (LPS) biosynthesis (Fig. S3C). In other cases, we simply observed slower growth, as in the case of *lptA* (involved in LPS transport) and *parC* (a subunit of topoisomerase). The contrast between *lpxC* and *lptA* phenotypes, both involved in LPS synthesis and transport, potentially suggests that *lpxC* may be the more vulnerable, more attractive target in the LPS pathway, although the degree of target KD was greater in *lpxC*-CRISPRi (−7 L2FC) than *lptA*-CRISPRi (−4 L2FC). Moreover, even within the same complex, we observed widely disparate growth phenotypes with similar levels of depletion, suggesting differential vulnerabilities even within the same complex, as previously observed in Mycobacteria within the DNA replication-related enzyme complexes (17) For example, we observed variable growth defects when we knocked down the terminal components of the essential LPS transport complex, which is composed of LptD and LptE, which together form the outer membrane, transport channel, and LptA, which forms the periplasmic bridge through which LPS is transferred before the LptD/E complex delivers it to the outer surface. Despite similar levels of target depletion in *lptD* and *lptA* (L2FC) of −4.27 and −3.67, respectively, relative to induced control), the degree of growth defect was more pronounced in *lptA*-CRISPRi^R^ than in *lptD*-CRISPRi^R^ (Fig. S3B and S3D). Further, despite KD of L2FC of −3, *lptE*-CRISPRi^R^ had no growth defect at all, phenocopying what has been observed in an arabinose-inducible *lptE* KD strain (33) (Fig. S3B and S3D). Similarly, while depletion of both *gyrA* and *gyrB* (L2FC of −4.4 and −2.9, respectively) of the DNA topology gyrase enzyme had minimal impact on growth, depletion of the *parC* subunit (L2FC of −3.4) of the related topoisomerase IV enzyme led to a dramatic growth defect (Fig. S3B and S3D). Thus, perturbing essential genes across similar processes (cell envelope modulating *murA* and *lpxC*), within the same pathway (*lptA*, *lptD*, and *lptE*), or having similar functions (*gyrA* and *parC*) led to diverse growth outcomes, which implies differential target vulnerability that has important implications for drug discovery efforts (17).

### CRISPRi to probe mechanism of action of small-molecule inhibitors

**Fig 4:**
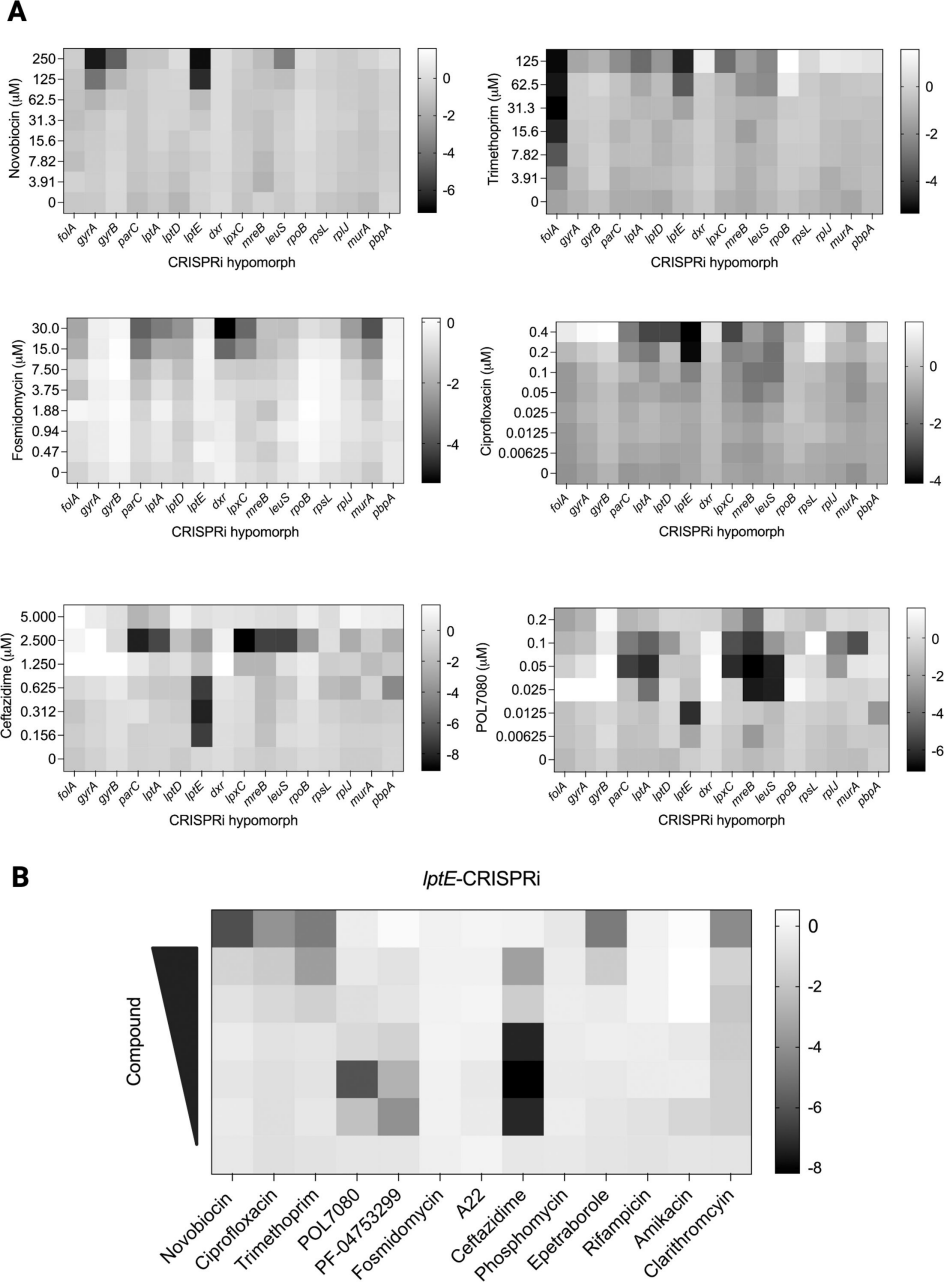
CRISPRi^R^ genetic perturbation leads to altered chemical sensitivities. (**A**) Heat map of the L2FC in growth of CRISPRi^R^ strains relative to the control-CRISPRi^R^ strain. CRISPRi^R^ strains were grown in induced conditions albeit with sub-lethal concentrations of rhamnose. (0.05% rhamnose: *folA*-, *gyrA*-, *gyrB*-, *lptE*-, *rpoB*-, and *pbpA*-CRISPRi^R^ strains; 0.025% rhamnose: *lptA*-, *lptD*-, *dxr*-, *mreB*-, *leuS*-, *rpsL*, and *rplJ*-CRISPRi^R^; 0.0125% rhamnose: *lpxC*- and *murA*-CRISPRi^R^.) Growth (OD_600_) was measured for each strain after 24 h in the presence of chemical inhibitor, and L2FC of growth relative to the growth of the control-CRISPRi^R^ strain was calculated. CRISPRi^R^ strains displayed variable sensitivities to chemical inhibitors. (**B**) Heat map of the L2FC in growth of the *lptE*-CRISPRi^R^ strain relative to control-CRISPRi^R^ strain reveals broad sensitization to a number of chemical inhibitors with different mechanisms of action (MOA). Scale indicates L2FC in growth relative to control-CRISPRi^R^ strain.

We used the tightly regulated CRISPRi^R^ system first to create partial KD mutants to identify chemical genetic interactions between specific genes with anti-pseudomonal molecules and then to generate maximal KD mutants to measure specific transcriptional signatures in response to pronounced essential target depletion. We first leveraged the tunability of the rhamnose CRISPRi system to examine chemical genetic interactions between the depletion mutants and small-molecule inhibitors (34–37). We used sub-lethal rhamnose doses to achieve partial gene depletion of the 16 selected essential genes and measured the activity of 13 known small molecules targeting DNA replication, transcription, protein translation, and cell envelope synthesis in these strains using standard growth-based assays (Fig. S5). We observed sensitization of a CRISPRi^R^ mutant to a chemical inhibitor in 12 of 13 cases (Fig. S6). For example, KD of *gyrB*, *folA*, and *dxr* (involved in isoprenoid biosynthesis) sensitized only to their respective inhibitors, novobiocin, trimethoprim, and fosmidomycin (Fig. 4A). Of note, KD of the other gyrase subunit, *gyrA*, also caused novobiocin sensitization, even though it does not bind GyrA directly, revealing a functional interaction between the two subunits in the target protein complex. None of the DNA synthesis-related CRISPRi^R^ strains showed shifts in susceptibility to ciprofloxacin, which directly binds GyrA and ParC in complex with DNA to stabilize DNA breaks (Fig. 4A) (38).

Meanwhile, in the case of cell wall active agents, we often observed chemical genetic interactions linking the cell wall and membrane integrity. For example, the β-lactam ceftazidime was not only more potent against the canonical penicillin-binding protein strain, *pbpA*-CRISPRi^R^, relative to wild-type, but also against membrane integrity-depleted strains such as *lpxC*- (involved in LPS biosynthesis), *lptA*-, *lptE*-, and *mreB*-CRISPRi^R^. Similarly, the cyclic peptide LptD inhibitor, POL7080, not only had enhanced potency toward its target LPS pathway-specific strains such as *lptA*-, *lptE*-, and *lpxC*-CRISPRi^R^ but also toward peptidoglycan synthesis-linked *mreB*-CRISPRi^R^ (Fig. 4A). Ceftazidime and POL7080 sensitivity profiles indicate crosstalk among genes involved in maintaining the cell envelope. Interestingly, POL7080 also had an interaction with the protein synthesis-related *leuS*-CRISPRi strain, potentially linking mistranslation and envelope stress (39). Alternatively, this observed interaction with *leuS*-CRISPRi could be due to the operon structure with *lptE* being immediately downstream of *leuS*, resulting in *lptE* depletion in the *leuS*-CRISPRi^R^ strain (*lptE* L2FC of −2.6 in the *leuS*-CRISPRi strain, Table S1). Notably, although the *lptE*-CRISPRi^R^ strain was not growth-impaired, it was broadly sensitized across the drug panel, indicating a vital role for LptE in maintaining the cell envelope barrier in *P. aeruginosa* to certain chemical inhibitors (Fig. 4B). For example, barrier disruption through *lptE* KD in the *lptE*-CRISPRi strain and from the polar effect on *lptE* seen in the *leuS*-CRISPRi strain led to sensitization to some large, structurally complex inhibitors like novobiocin and clarithromycin but not amikacin. KD strains of *lptE* were also sensitized to the inhibition of peptidoglycan crosslinking by ceftazidime but not to inhibition of peptidoglycan synthesis by phosphomycin. Thus, growth inhibitor sensitization studies with CRISPRi-based hypomorphs showed direct as well as more complex chemical genetic interactions, which can provide insights into gene/pathway functions in *P. aeruginosa*.

### Expression profiling on CRISPRi strains sheds light on the *P. aeruginosa* response to genetic perturbations

We also leveraged the rhamnose-based system to achieve maximal KD of essential genes in *P. aeruginosa* in order to measure genome-wide transcriptional responses to specific genetic depletions. These target-specific transcriptional changes were then mapped to the equivalent changes due to chemical perturbation of the corresponding essential pathways, utilizing our previously reported PerSpecTM method (40). We initially performed transcriptomic analysis of the 16 CRISPRi^R^ KD strains after 6 h of induction with maximum concentrations of rhamnose, as previously performed using the arabinose CRISPRi^A^ system. However, we noted that the degree of transcriptional change in induced strains relative to control-CRISPRi strains was less for rhamnose-based strains than for arabinose ones at this same time point (Fig. 2E). We thought this could be due to the relatively shorter induction period of the rhamnose system, taking into account that the leak in the arabinose system would have provided longer, sustained activation of the system (Fig. 2E). When we extended the period of rhamnose induction to 10 h, we measured similar levels of target gene KD as at 6 h but were able to capture broader genome-wide responses to perturbation, based on the number of differentially expressed genes (Fig. S7A and S7B).

**Fig 5:**
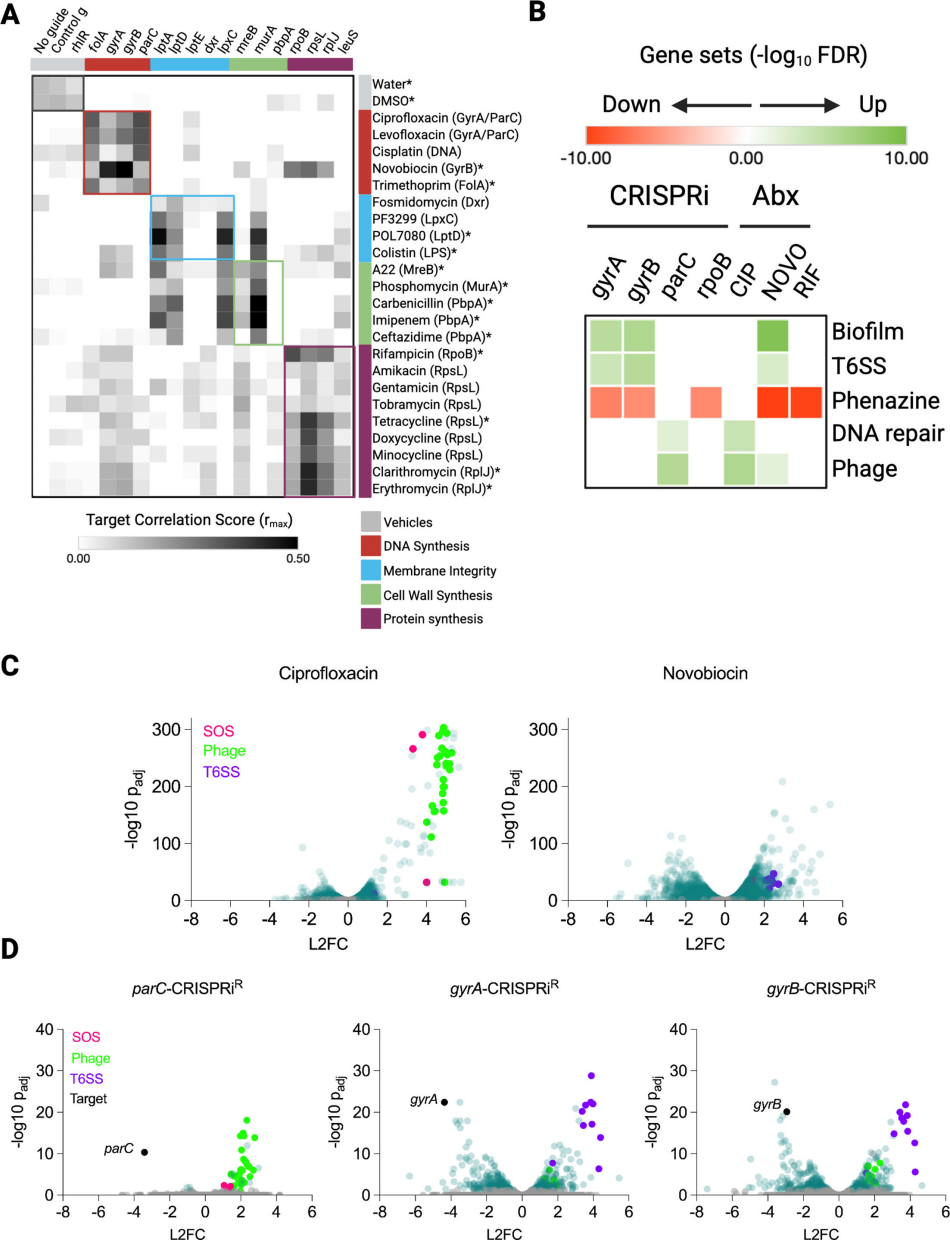
Transcription profiling of CRISPRi^R^ KD strains reveals chemical genetic interactions. (**A**) PerSpecTM analysis of chemical inhibitors queried against CRISPRi^R^ strains. Pearson correlations (${\overset{-}{r}}_{max}$) of z-scored expression profiles from 90-min chemical inhibitor treatment on PA14 with 10 h of gene depletion of CRISPRi^R^ strains are shown as a heat map. Responses to all chemical inhibitors were characterized in PA14 except fosmidomycin, which was characterized in a dxr-hypomorph strain due to the lack of wild-type activity. CRISPRi strains and chemical inhibitors with the same or related MOA are color-coordinated. The transcription profiles of chemical inhibitors largely cluster according to their MOA. Cell envelope genetic perturbations show a degree of crosstalk between membrane integrity and peptidoglycan synthesis chemical inhibitors. The asterisk indicates higher Pearson correlations between transcription profiles of a chemical inhibitor and genetic perturbation of its predicted target with CRISPRi^R^ than with CRISPRi^A^ (Fig. S7) (40). (**B**) Heat map showing gene sets enriched after CRISPRi^R^ genetic perturbation relative to the control-CRISPRi^R^ strain or after chemical perturbation relative to vehicle control. Enrichment is quantified by the false discovery rate measured using STRING-DB, with green and red colors indicating upregulated and downregulated gene sets, respectively. White boxes indicate no functional enrichment of a gene set. CRISPRi^R^ perturbation revealed a distinct bacterial response to gyrase versus topoisomerase IV depletion. The DNA damage response (SOS) leading to upregulation of DNA repair and phage assembly genes, which is a hallmark of fluoroquinolone exposure, was observed after topoisomerase IV knockdown (*parC*-CRISPRi^R^) but not gyrase (*gyrA*-, *gyrB*-CRISPRi^R^) nor RNA-polymerase (*rpoB*-CRISPRi^R^) KD. (**C**) Volcano plots showing gene expression in PA14 in response to ciprofloxacin and novobiocin treatment. Ciprofloxacin-induced genes involved in SOS response (pink) and phage assembly (blue). (**D**) Volcano plots showing gene expression in response to CRISPRi^R^ KD of topoisomerase genes. *parC* KD elicited upregulation of SOS response and phage assembly genes, whereas *gyrA* and *gyrB* KD led to type VI secretion system (T6SS) upregulation.

Previously, PerSpecTM analysis of a large *P. aeruginosa* transcriptomic data set accurately predicted the MOAs of known and novel small-molecule inhibitors (40). Predictions were made based on aligning inhibitor-induced bacterial transcriptional responses with reference profiles from known-MOA compounds and from arabinose-inducible target KDs. Compounds sharing a mechanism showed correlated transcriptional signatures. Notably, the transcriptional response to a small molecule could also be correlated with the profile resulting from genetic KD of its cognate target. Here, using CRISPRi^R^ strains with the rhamnose-inducible KD after 10 h of induction, we observed stronger Pearson correlations between gene KD profiles and their corresponding small-molecule treatments than with profiles generated using the arabinose-based system for 15 of 25 chemical perturbations (Fig. 5A; Fig. S8). Moreover, matching transcriptional profiles between small molecules and their CRISPRi-depleted target strains proved more sensitive for identifying target relationships than mapping small molecules to target genes based on CRISPRi hypersensitivity growth phenotypes. For example, while strains depleted of *rpoB*, *rpsL*, and *rplJ* (which encode subunits of RNA polymerase, the 30S ribosome, and 50S ribosome, respectively) were not hypersensitized to transcription or translation inhibitors (rifampicin, tetracyclines, or macrolides) in a growth-based MIC_90_ assay (Fig. S6), a robust interaction emerged based on correlation of transcriptional profiles for *rpoB*-CRISPRi with rifampicin treatment and *rpsL*-CRISPRi and *rplJ*-CRISPRi strains with treatment by two protein synthesis inhibitor classes (Fig. 5A). Similarly, while we saw no sensitization of any of the DNA synthesis-associated CRISPRi^R^ strains to ciprofloxacin (Fig. S6), the transcriptional responses to fluoroquinolones and cisplatin were significantly correlated with the response to *parC* KD (Fig. 5A). These observed correlations demonstrate the greater sensitivity of a gene expression readout relative to an OD_600_-based growth measurement for identifying chemical genetic interactions.

While molecule-molecule, molecule-gene, or gene-gene correlations were based on the genome-wide patterns of transcriptional response, agnostic to specific gene identities, the actual genes and pathways whose expression is perturbed by a molecule or gene KD can provide insights into a molecule’s MOA or a gene’s function. We performed differential gene expression analysis followed by functional enrichment using STRING-DB to identify pathways that were differentially induced in response to specific perturbations (Fig. 5B). Interestingly, the SOS response—a hallmark DNA damage and repair—was induced when *parC* but not *gyrA* or *gyrB* were depleted, suggesting that reduction in ParC’s decatenation activity results in accumulation of single-stranded DNA breaks, double-stranded DNA breaks, or unresolved replication intermediates that trigger the RecA-mediated SOS response. Key SOS genes such as *recA*, *recN*, and phage-related genes downstream of the SOS response were among the most highly upregulated upon *parC* depletion or from ciprofloxacin treatment but not novobiocin treatment (Fig. 5C and D). In contrast, the loss of gyrase’s negative supercoiling activity alone in the *gyrA-* and *gyrB-*depleted strains, *per se*, did not cause sufficient DNA damage to induce the SOS response (Fig. 5D). Further, in that fluoroquinolones are highly activating of the SOS response, these results are consistent with what has now been long established, that fluoroquinolones do not act simply by inhibiting enzymatic function at least in the case of gyrase; instead, they act by a poisoning mechanism (38, 41). This result is in contrast to novobiocin, which is not only more potent against the *gyrB-* and *gyrA-*depleted strains (Fig. 4A) but also phenocopied the transcriptional consequences of *gyrB* and *gyrA* depletion (Fig. 5A). These results together point to a simple enzymatic inhibition mechanism for novobiocin.

Together, these findings show that CRISPRi-mediated KD often mimics the transcriptional effects of chemical inhibition of essential pathways. Moreover, some KDs reveal unique response signatures, helping link specific gene functions to stress responses in *P. aeruginosa*.

## DISCUSSION

We present an improved CRISPRi system using the *S. pasteurianus* dCas9 for *P. aeruginosa* with enhanced control over gene KD, enabling robust genetic perturbation studies of essential genes. In contrast to a recently reported CRISPRi doxycycline-induced *S. pyogenes* dCas9 system for *P. aeruginosa* (14), we focused on small hydrophilic, sugar-based induction systems as they are less susceptible to efflux pumps, thereby increasing the likelihood that they could be applied broadly to *P. aeruginosa* strains including clinical isolates, which generally have high baseline efflux pump activity and might show less mutant-to-mutant variability in inducer cell entry and intracellular accumulation. Building on a prior system utilizing *S. pasteurianus* dCas9 (dCas9_Spa_), we optimized expression control to target essential genes not only to ensure robust target repression but also, importantly, to ensure no activation in the absence of inducer to ensure stable maintenance of individual mutants or pools of mutants with propagation. This feature is critical for enabling studies of essential gene function. In contrast to our arabinose-inducible CRISPRi^A^ system and other reported sugar-based systems relying on arabinose-induction (P_araBAD_) or IPTG-induction (P_lac/tac_) of dCas9 with constitutive promoters driving sgRNA expression (13, 15), which resulted in unintended gene repression in the absence of inducer, we employed a different approach. We used the rhamnose-inducible P_rhaBAD_ promoter to regulate both *dcas9* and sgRNA expressions, inspired by the tightly regulated CRISPRi system in *Mycobacterium spp* (16). To accommodate the larger P_rhaBAD_ promoter, we divided the CRISPRi system across two integrating vectors: one expressing dCas9 for integration into the *ϕ CTX* attachment site, thereby generating a parental CRISPRi strain (PA14::*dcas9*), and the other expressing sgRNAs for integration into the *att*Tn7 site (Fig. 2A). This modular design enabled efficient cloning and integration via conjugation of compact sgRNA-carrying miniTn7 plasmids into PA14::*dcas9*, unlike the inefficient integration of a single, large dual-function plasmid (Fig. 1A). The combination of tight regulation of both *dcas9* and sgRNA from the rhamnose promoter and high conjugation efficiency facilitated rapid and facile—even pooled—construction of CRISPRi KD libraries in *P. aeruginosa* (Fig. S4B). We also identified a new PAM sequence, NNGTAT, which in combination with the three known PAMs will expand the scope of guide locations in the target genome, further enabling genome-wide perturbation.

Using our dCas9_Spa_-based system, we knocked down 16 essential genes involved in diverse cellular processes, including DNA replication, transcription, translation, and envelope biogenesis. As expected, we observed highly variable phenotypes depending on the gene and the degree of KD that could be achieved, based on the vulnerability of each gene, i.e., its ability to tolerate depletion before impacting the phenotype. For instance, *lpxC* depletion, which impacts LPS synthesis, was bactericidal, whereas *murA* KD, which impacts peptidoglycan synthesis, was bacteriostatic, highlighting differential phenotypes across envelope biogenesis pathways. Moreover, within the same complex, similar levels of depletion of different components resulted in widely disparate growth phenotypes, as previously described in Mycobacteria (17) and here exemplified in depletion of Lpt complex components *lptD*, *lptA*, and *lptE* (Fig. S3D). Assuming the relative depletion of their mRNA levels translates to protein depletion, these differences suggest that LptA may be the most vulnerable target in the pathway and might be the stoichiometric bottleneck for Lpt complex formation and function (42, 43). Similarly, among DNA topology-modulating type II topoisomerase enzymes, *parC* depletion caused severe growth defects, while depletion of gyrase genes, *gyrA* and *gyrB*, had only modest effects (Fig. S3D).

For a given gene, we typically observed changes in the transcription profile occurring even with low levels of depletion as the cell may sense stress or imbalance and attempt to compensate. With greater depletion, increased sensitivity to inhibitors of the target became manifest because of the less available functional target. Only with extreme levels of depletion where there is no longer sufficient functional target for normal viability did we observe a detectable impact on survival and growth (44, 45). Thus, changes in transcriptional programs, followed by MIC_90_ shifts, and then finally growth defects formed a typical hierarchy of sensitivity to target depletion. This hierarchy was demonstrated by a number of genes in this study. For example, in the case of *folA* and *mreB*, the corresponding uninduced arabinose-based strains had no growth defects but did display shifts in MIC_90_ to cognate inhibitors or morphologic changes, reflecting more sensitive detection of depleted function (Fig. 1D and E). Further, while strains depleted of *rpoB*, *rpsL*, *rplJ*, and *parC* were not hypersensitized to cognate inhibitors of the corresponding gene product or pathway in a growth-based MIC_90_ assay (Fig. S6), the transcriptional profiles resulting from genetic depletion correlated well with treatment of wild-type PA14 by their corresponding cognate inhibitor (Fig. 5A).  

Comparisons of these phenotypes in different genetically depleted strains can reveal novel aspects of gene function, particularly when discordance is detected among similar targets, such as those within the same complex. For example, while *lptE* depletion did not impair growth, KD sensitized (4- to 8-fold shift in MIC_90_) to several anti-pseudomonal molecules including POL7080 and PF-04753299, which target LPS synthesis; novobiocin and clarithromycin, which are typically excluded by the *P. aeruginosa* outer membrane; and ceftazidime, the β-lactam peptidoglycan crosslinking inhibitor. We also observed moderate sensitization (2- to 4-fold shift in MIC_90_) to ciprofloxacin, trimethoprim, and epetraborole. These results suggest that despite LptE’s relative invulnerability to depletion with respect to growth impairment, it potentially plays a unique role in outer membrane integrity, relative to other, more growth-vulnerable Lpt complex proteins such as LptA, which may serve as the stoichiometric determining subunit of the Lpt complex (46–48).

Similarly, *parC* KD differed from *gyrA* and *gyrB* depletion, with the former inducing the SOS response including DNA repair and phage assembly pathways, which is the hallmark bacterial response to DNA breaks, thereby phenocopying the response to fluoroquinolone exposure. In contrast, a bit surprisingly, the gyrase depletion of both *gyrA* and *gyrB* subunits resulted in the strong upregulation of biofilm genes and the T6SS and repression of quorum sensing genes in the PQS pathway, without activation of the SOS response (49). The mechanism underlying this surprising relationship between gyrase depletion and these virulence mechanisms remains to be elucidated. Nevertheless, these divergent transcriptional responses to depletion underscore fundamental differences in the mechanisms by which *P. aeruginosa* senses and responds to disruption of gyrase, topoisomerase IV, and/or their regulatory pathways. Further, they highlight the complexity of fluoroquinolone activity in contrast to novobiocin activity, with the latter acting as a direct inhibitor of GyrB enzymatic function while fluoroquinolones act via a topoisomerase poisoning mechanism. Thus, genetic perturbation can result in phenotypic consequences distinct from growth to reveal new insights into gene function or small-molecule MOA (40).

While the application of CRISPRi to this set of 16 genes provided insights into some chemical interactions and target vulnerabilities, this study nevertheless was limited in scope. Phenotypic outcomes are dependent on the degree of KD achieved and the functional reserve of a target, i.e*.,* ability of the cell to tolerate target depletion. In the case of CRISPRi, the nature of the selected sgRNA, including PAM strength, guide location, and guide length, is an important factor that determines target KD levels, and consequently, cellular responses. The other important factor determining phenotypic outcomes is the inherent vulnerability of the target gene. Although we attempted to select the strongest PAM for each gene in order to elicit the most dramatic phenotypes approximating complete target depletion, based on the mKate2 reporter assay and guide position closest to the transcription start site (Fig. S2), it is possible that we did not in reality achieve this goal in all cases. Thus, suboptimal guides resulting in insufficient or variable target depletion could explain some of the differential growth and inhibitor susceptibility profiling phenotypes that we observed. To identify the strongest sgRNA in an unbiased manner, a more comprehensive pooled CRISPRi KD library, which includes multiple sgRNAs across a range of strengths, would need to be systematically evaluated (17). A related caveat in assessing the level of target KD by CRISPRi is that mRNA KD levels do not always accurately reflect the extent of protein depletion; this distinction is important to consider when interpreting the relationship between genetic vulnerability and cellular phenotype.

Another limitation of CRISPRi is that it can generate polar effects in bacterial systems, including in *P. aeruginosa*, due to the operonic organization of bacterial genomes. Thus, the targeted KD of one gene could also impact genes downstream in the operon, leading to effects on observed phenotypes that are not due to the specific CRISPRi targeted gene. We observed such effects with some of our targets, like *mreB*, wherein we observed downregulation of downstream *mreC* and *mreD* in *mreB*-CRISPRi^R^, and potentially *lptE* in *leuS*-CRISPRi^R^*,* which lies downstream of *leuS*. However, clearly, this is not a universal phenomenon since in other CRISPRi strains, such as *lptA-*CRISPRi^R^, with the *lptB* gene located downstream, we did not observe any polar effects. Therefore, large-scale CRISPRi-based functional or phenotypic characterization needs to include controls that account for these polar effects. Despite all of the caveats in using CRISPRi to study the consequences of genetic depletion, we nevertheless found that the observed phenotypes consistently aligned with expected target depletion effects.

In conclusion, the rhamnose-inducible CRISPRi system enables precise and tunable repression of essential genes in *P. aeruginosa*, thus making it well-suited both for detailed analysis of individual essential genes and for large-scale, genome-wide functional genomic studies and chemical-genetic screens—approaches that have already proven powerful in other bacterial pathogens such as *E. coli* (50–54), *B. subtilis* (11, 55), mycobacteria (17, 56), and most recently *P. aeruginosa* (14). Notably, this system supports the generation and stable maintenance of comprehensive pooled mutant libraries inclusive of essential genes, agnostic of strain efflux pump background, thereby allowing for comprehensive, systematic assessment of individual gene vulnerabilities across a large number of clinical isolates. Such studies will provide critical insights for target prioritization in antibiotic discovery efforts and will help accelerate the development of new therapeutics against *P. aeruginosa*, a pathogen that has proven historically recalcitrant to traditional antibiotic discovery strategies.

## MATERIALS AND METHODS

### CRISPRi cloning and strain construction

All sgRNA sequences and primer information are shown in Table S1.

#### First-generation P_araBAD_ CRISPRi^A^ system

The catalytically inactive Cas9 (dCas9) from *Streptococcus pasteurianus* and *Streptococcus pyogenes* was purchased from Addgene (Watertown, MA) (13, 15); the dCas9 from *Streptococcus thermophilus* was gifted by Jeremy Rock (16). The *dcas9* gene was cloned into a modified pUC18T-miniTn7T-pBAD-Gm vector previously described by our group under the control of the AraC-araBAD promoter system using standard 2-piece Gibson Assembly (57). Three BsmBI restriction sites were removed by the QuikChange XL mutagenesis kit (Agilent, Santa Clara, CA). A custom gene block was synthesized containing a 5′ HindIII restriction site, *E. coli* ProD promoter, two unique BsmBI restriction sites positioning the sgRNA at the transcriptional start site of ProD promoter, rrnB T1 terminator, and a KpnI restriction site. The gene block and pUC18T-miniTn7T-pBAD-dcas9-Gm plasmid were restriction-digested with HindIII and KpnI and ligated together (T4 DNA ligase, New England Biolabs, Ipswich MA) to produce the first-generation CRISPRi^A^ plasmid pUC18T-miniTn7T-pBAD-dcas9-pD-Gm (9,326 bp). sgRNAs were cloned into the CRISPRi^A^ plasmid using standard CRISPRi protocols established in mycobacteria using BsmBI cut sites (16). Another iteration of the CRISPRi^R^ dCas9_Spa_ was constructed on a pUC18-miniTn7T-pRha-dcas9-pD-Gm vector under the control of the RhaRS-rhaBAD promoter system (10,140 bp). All constructs were transformed into 10-beta *E. coli* (New England Biolabs, Ipswich MA). Following rapid plasmid sequencing, tetrapartite bacterial conjugation was carried out in a 1:1:1:1 mixture of recipient (PA14), 10-beta *E. coli* donor cells harboring the construct of interest, and *E. coli* cells harboring the helper plasmids pRK2013 and pTNS2 (24). Recipient PA14 cells were incubated at 42°C for 2 h prior to the tetrapartite mating. Cells were mated at 37°C for 24 h on cellulose membranes, resuspended in 400 μL of LB, and plated on LB agar containing 15 μg/mL gentamicin and 5 μg/mL irgasan. Colonies were screened for correct integration of the miniTn7 plasmid using primers 10 and 12.

#### Constitutive mkate2 strain used with first-generation CRISPRi^A^

The fluorescent gene *mkate2* was custom-synthesized as a gene block under the control of a constitutive P_trc_ promoter. Gene blocks of *mkate2* with mutated PAM sites were also synthesized. The ptrc-mkate2 fragment was polymerase chain reaction (PCR) amplified using primers 1 and 2 and double-digested with SpeI and SacI. The digested fragment was ligated into the double-digested miniCTX1 (58) plasmid and transformed into 10-beta *E. coli*. Following rapid plasmid sequencing, tripartite bacterial conjugation was carried out in a 1:2:2 mixture of recipient (PA14), 10-beta *E. coli* donor cells harboring the CTX-*mkate2* construct, and *E. coli* cells harboring the helper plasmid pRK2013. Recipient PA14 cells were incubated at 42°C for 2 h prior to the tripartite mating. Cells were mated at 37°C for 24 h on cellulose membranes, resuspended in 400 μL of LB, and plated on LB agar containing 15 μg/mL gentamicin and 5 μg/mL irgasan.

#### Second-generation P_rhaBAD_ CRISPRi^R^ system

The second-generation system split the CRISPRi components into two plasmids: moving the *dcas9* gene with a rhamnose-inducible promoter on a miniCTX1 vector to create a PA14:dcas9 strain that can be used as the recipient strain for any guide and moving the sgRNA with a rhamnose-inducible promoter on the smaller pUC18-miniTn7T-Gm plasmid (RhaBAD vector: 6,470 bp) to facilitate cloning and conjugations. The miniCTX1-ptrc-mkate2-tet (see constitutive mkate strain above) and the pUC18-miniTn7T-pRha-dcas9-pD-Gm vectors were double-digested with HindIII and SacI to excise the ptrc-mkate fragment and RhaRS-rhaBAD-dcas9 fragment, respectively. Linearized miniCTX1 and the RhaRS-rhaBAD-dcas9 fragment were ligated together to create a miniCTX1-pRha-dcas9-Tet plasmid (11,874 bp). Following transformation into 10-beta *E. coli* and rapid plasmid sequencing, the plasmid was conjugated into PA14 using the tripartite mating strategy. Positive conjugants were selected on 100 μg/mL tetracycline and 5 μg/mL irgasan and screened using primers 5 and 6 to check for correct CTX integration (58) at the *glmS* site and primers 3 and 4 to check for the presence of *dcas9*. To remove the tetracycline resistance cassette in the FRT region of the integrated miniCTX1 plasmid, a clone of PA14:CTX-dcas9 was mixed in a 1:5 ratio with *E. coli* cells carrying the SM10-pFLP2 flippase plasmid and incubated at 37°C for 24 h on cellulose membranes. Conjugants were selected on LB agar 5 μg/mL irgasan. After overnight incubation at 37°C, colonies were patched onto LB agar with 5 μg/mL irgasan, and LB agar with 100 μg/mL tetracycline + 5 μg/mL irgasan. After overnight incubation at 37°C, colonies that grew on LB 5 μg/mL irgasan and not 100 μg/mL tetracycline were streaked onto LB agar with 15% sucrose to remove the SM10-pFLP2 plasmid. Single colonies that grew overnight at 37°C were patched onto LB 100 μg/mL carbenicillin and LB 100 μg/mL tetracycline to check for the loss of the SM10-pFLP2 plasmid and loss of the tetracycline cassette in the FRT region. Positive clones were screened by PCR to confirm the absence of the FRT region and presence of RhaBAD-dcas9 using primers 5 and 7 and 3 and 4, respectively.

#### Second-generation sgRNA plasmid

A Tn7 vector with a rhamnose-inducible promoter controlling sgRNA expression was constructed to facilitate the cloning of sgRNAs into the parental PA14::*dcas9* strain. Q5 mutagenesis was performed on the pUC18-miniTn7T-pRhabad-dcas9-pD-Gm plasmid to remove the *dcas9* coding sequence and ProD promoter and bring the Rhabad promoter transcriptional start site in line with the sgRNA generating pUC18-miniTn7T-pRhadbad-Gm. Constructs were transformed into 10-beta *E. coli* and rapid plasmid-sequenced. sgRNAs were cloned into the CRISPRi^R^ plasmid using standard CRISPRi protocols established in mycobacteria using BsmBI cut sites (16). Sequence-confirmed sgRNA plasmids were conjugated into PA14:CTX-dcas9 (PA14::*dcas9*) parental strain using the tetrapartite method described above. Positive conjugants were selected on LB agar with 15 μg/mL gentamicin and 5 μg/mL irgasan and PCR-screened for correct *attB*-Tn7 integration using primers 10 and 12.

#### Constitutive mkate2 strain used with second-generation CRISPRi^R^

The miniCTX-ptrc-mkate plasmid was double-digested with BamHI and SacI to remove the ptrc-mkate fragment. The recipient sgRNA vector pUC18-Tn7T-pRhabad-Gm was also double-digested and ligated with the ptrc-mkate fragment. Constructs were used to clone in *mkate2* guides using BsmBI cut sites and conjugated into PA14::*dcas9* parental strain to test the efficacy of the P_rhaBAD_ CRISPRi^R^ system.

### dCas9 Western blot

Overnight cultures grown in LB (37°C, 250 rpm) were diluted 100-fold in fresh LB with or without arabinose (0.5%) and grown until OD_600_ reached mid-log phase. Twenty milliliters of cells was pelleted and resuspended in 1 mL of lysis buffer (50 mM Tris, pH 7.5, 1 mM EDTA, 150 mM NaCl, 5 μg/mL DNase I, 0.5 mg/mL lysozyme, and Roche protease inhibitor cocktail). Cells were lysed by sonication and supernatants clarified by centrifugation at 13,000 g at 4°C for 10 min. Protein concentrations were quantified using the micro-BCA assay. Protein extracts (20 μg) were separated by SDS-PAGE (4%–20% Biorad TGX gels) and transferred to polyvinylidene fluoride membranes. Membranes were incubated overnight with primary anti-6×his mouse (Sigma #SAB2702218) in a 1:5,000 dilution or anti-RpoA mouse (Sigma #F3165) in a 1:20,000 dilution at 4°C. Membranes were washed and incubated with anti-mouse HRP at 1:10,000 dilution for 1 h at room temperature.

### mKate2 fluorescence KD

Overnight cultures grown in LB (37°C, 250 rpm) were diluted 100-fold in M9 minimal media (M9 salts, 0.4% glucose, 2 mM MgSO_4_, and 0.1 mM CaCl_2_) and grown until OD_600_ reached mid-log phase. Cultures were diluted to OD_600_ 0.01 and added to a 96-well plate (Nunc 96-well black, optical bottom plate, Thermo Fisher Scientific, Waltham MA) containing the appropriate amount of arabinose/rhamnose. The plate was incubated in a humidity chamber with shaking at 37°C in a Spark Multimode Reader (Tecan, Männedorf, Switzerland), where fluorescence (Ex 588 nm, Em 633 nm) and OD_600_ were measured every hour for 24 h. PA14 was used to subtract background fluorescence. First-generation CRISPRi^A^ *mkate2* KD strains were compared to a control-CRISPRi^A^ strain that carried a scrambled control sgRNA that had no impact on fluorescence or growth in minimal media. Second-generation CRISPRi^R^ *mkate2* KD strains were compared to a no sgRNA control strain that carried a guide-less pUC18-Tn7T-ptrc-mkate-pRhabad-Gm plasmid.

### CRISPRi growth kinetics and minimum inhibitory concentration measurements

#### Growth in liquid

Overnight cultures grown in LB (37°C, 250 rpm) were diluted 100-fold in fresh LB or with LB-arabinose or rhamnose to pre-activate the system and grown until OD_600_ reached mid-log phase. Cultures were diluted to OD_600_ 0.001 and 25 µL added to a 384-well plate (Nunc 384-well clear polystyrene plates, Thermo Fisher Scientific, Waltham MA) containing the appropriate amount of 2× arabinose/rhamnose or chemical inhibitor. The plate was incubated in a humidity chamber with shaking at 37°C in a Spark Multimode Reader (Tecan, Männedorf, Switzerland) where OD_600_ was measured every hour for 24 h. Chemical genetic interaction profiles were calculated by the fold change in growth of the target-CRISPRi^R^ strain relative to the control-CRISPRi^R^ strain.

For kill kinetics experiments, cultures were diluted to OD_600_ 0.0001 in 15 mL glass tubes containing the appropriate amount of rhamnose. The tubes were incubated at 37°C 250 rpm for up to 12 h. One hundred microliter aliquots were collected, 10-fold serially diluted in 1× PBS, and plated on LB agar in 10 µL spots. Plates were incubated overnight at 37°C in a humidity box. Colonies were counted and plotted as colony-forming unit (CFU)/mL.

#### Growth on solid media

Overnight cultures grown in LB (37°C, 250 rpm) were diluted 100-fold in fresh LB and grown until OD_600_ reached mid-log phase. One hundred microliter aliquots were collected, 10-fold serially diluted in 1× PBS, and plated on LB agar with the appropriate amount of arabinose in 10 µL spots. Plates were incubated overnight at 37°C in a humidity box.

### Microscopy

Overnight cultures grown in LB (37°C, 250 rpm) were diluted 100-fold in fresh LB and grown until OD_600_ reached mid-log phase. Cultures were diluted to OD_600_ 0.001 and 75 µL added to a 96-well plate with 75 µL of the corresponding 2× rhamnose or arabinose and incubated for 19 h. One hundred microliters of cells was collected and mixed with 100 µL 1× PBS and spun down at 9,000 rcf for 10 min. The supernatant was removed and the pellet suspended in 50 µL 1× PBS with 2.5 µg/mL of FM-143x (Invitrogen #F35355). Samples were incubated on ice for 15 min. One hundred fifty microliters of 1× PBS was added on top, pelleted, and resuspended in 50 µL 4% paraformaldehyde in 1× PBS. Samples were incubated on ice for 20 min. One hundred fifty microliters 1× PBS was added on top, pelleted, and resuspended in 20 µL 1× PBS. One microliter of stained cells and 3 µL of ProLong Gold Antifade reagent (Invitrogen #P36930) were added to a glass microscope slide, covered with a coverslip, and imaged on a Zeiss confocal microscope after 24 h of drying at 63× magnification with a 2× digital zoom using a 488 nm laser and Cy3 acquisition filter.

### RNA-Seq experiments

RNA-Seq experiments were carried out in biological triplicate in 384-well microplates (Nunc 384-well clear polystyrene plates, Thermo Fisher Scientific, Waltham MA). Overnight cultures of CRISPRi^R^ strains grown in LB (37°C, 250 rpm) were diluted 50-fold in fresh LB in a 96 deep-well block. Bacterial cultures were grown with shaking (37°C, 250 rpm) to mid-log phase, and 30 μL of cultures was mixed with equal volume of 2× rhamnose working solution, yielding final conditions containing 1 × 10^8^ CFU/mL bacteria and with 0%, 0.0125%, or 0.05% rhamnose in LB. CRISPRi^R^ strains were treated at 37°C without shaking in a humidity chamber for 6 h. After incubation, 50 μL of cultures were mixed with 25 μL 3× RNAgem Blue Buffer (Zygem, Charlottesville VA) and chemically lysed by incubation at 75°C for 10 min in a thermocycler. Total RNA was extracted and purified using the Direct-zol RNA Miniprep Plus kit and TRIzol reagent (Zymo Research, Irvin CA). RNA quality and quantity were analyzed using the RNA ScreenTape with 2200 TapeStation (Agilent, Santa Clara CA).

An alternative experimental setup was used for the kinetics experiment. After diluting the overnight cultures 50-fold in fresh LB in a 96-well block, cells were back diluted to OD_600_ ~0.05 in 96-well blocks with/without 0.05% rhamnose. Cells were grown until OD_600_ 0.4–0.6 was reached, 120 μL of culture was mixed with 3× RNAgem Blue Buffer (Zygem, Charlottesville, VA) and chemically lysed by incubation at 75°C for 10 min in a thermocycler. Remaining cultures were back-diluted to OD_600_ 0.1 in 96-well blocks with rhamnose maintained at 0% or 0.05%. Once the uninduced cultures reached OD_600_ 0.3, 120 μL from all samples was mixed with 3× RNAgem Blue Buffer (Zygem, Charlottesville, VA), and chemically lysed by incubation at 75°C for 10 min in a thermocycler.

### Generation of RNA-Seq data

Illumina cDNA libraries were generated using a modified version of the RNAtag-seq protocol (59). Briefly, 500 ng–1 μg of total RNA was fragmented, depleted of genomic DNA, dephosphorylated, and ligated to DNA adapters carrying 5′-AN_8_-3′ barcodes of the known sequence with a 5’ phosphate and a 3′ blocking group. Barcoded RNAs were pooled and depleted of rRNA using the RiboZero rRNA depletion kit (Epicenter). Pools of barcoded RNAs were converted to Illumina cDNA libraries in two main steps: (i) reverse transcription of the RNA using a primer designed to the constant region of the barcoded adapter with addition of an adapter to the 3’ end of the cDNA by template switching using SMARTScribe (Clontech) as described (60); (ii) PCR amplification using primers whose 5′ ends target the constant regions of the 3’ or 5’ adapters and whose 3’ ends contain the full Illumina P5 or P7 sequences. cDNA libraries were sequenced on the Illumina (NextSeq 500) platform to generate paired-end reads.

### Computational analysis of RNA-Seq data

Sequencing reads from each sample in a pool were demultiplexed based on their associated barcode sequence using custom scripts. Up to one mismatch in the barcode was allowed, provided it did not make assignment of the read to a different barcode possible. Barcode sequences were removed from the first read as were terminal G’s from the second read that may have been added by SMARTScribe during template switching.

### Pathway analysis of RNA-Seq data

DESeq2 (61) was used to calculate the differential gene expression (L2FC and adjusted p-values) for each CRISPRi strain relative to the control-CRISPRi strain at matched rhamnose dose or time point. Pathways enriched with significantly up- or downregulated genes with an adjusted *P*-value cutoff of 0.05 were identified using STRINGdb (62) and visualized using Morpheus (63).

### PerSpecTM analysis

Target prediction using the CRISPRi^R^ P_rhaBAD_ strains was performed as previously described (40). Briefly, z-scored gene count tables were used to calculate Pearson correlation values between CRISPRi^R^ strain KD and the PA14 reference strain treated with compound for 90 min. Gene expression profiles from fosmidomycin treatment used a *dxr*-hypomorph as fosmidomycin does not have wild-type PA14 activity. A target correlation score (${\overset{-}{r}}_{max}$) was calculated between the compound (as the query) and CRISPRi^R^ strain (as the reference set). The correlation scores were visualized using Morpheus.

### Genomic DNA extraction and Illumina sequencing

Genomic DNA (gDNA) was isolated from bacterial sweeps from pooled conjugation selective LB agar plates or from aliquots of culture from uninduced passaging of CRISPRi pools using the Qiagen DNeasy Blood & Tissue Kit (#69506). gDNA was quantified using the Qubit dsDNA kit (#Q32854, Invitrogen). The sgRNA region was amplified from 100 ng of gDNA using NEBNext Ultra II Q5 master mix (#M0544L, New England Biolabs, Ipswich, MA) and the following PCR conditions: 98°C for 45 s; 17 cycles of 98°C for 10 s, 64°C for 30 s, 65°C for 20 s; 65°C for 5 min. The sgRNAs were amplified using forward primer 13 (0.5 μM final concentration) that appends a P5 flow cell adapter sequence and a Read1 Illumina sequencing primer binding site and reverse primer 14 (0.5 μM final concentration) that appends a P7 flow cell adapter sequence and a Read2 Illumina sequencing primer binding site. Primers contain unique barcodes for sample pooling during Illumina sequencing. The ~230 bp amplicons were purified using AMPure XP beads (Beckman-Coulter #A63880) with one-sided selection (1.2×). Amplicon size, purity, and concentration were measured using D1000 DNA ScreenTape (#5067–5582, Agilent, Santa Clara, CA) on a 2200 TapeStation (Agilent, Santa Clara, CA). Amplicons from each sample were multiplexed sequenced with Illumina MiSeq 300 (paired-end 2 × 150 cycles, 1 × 8 i5 cycles, and 1 × 8 i7 cycles).

### Quantification of sgRNA

To measure the abundance of sgRNAs from the pooled conjugations or from the CRISPRi pools, sgRNA sequencing read pairs were processed using Trimmomatic (64) and aligned using subread-align (65). Trimmomatic clipped the bases before base 30 on the 5′ end and after base 80 on the 3′ end, generating ~50 bp reads. Processed 50 bp reads were aligned to the full-length reference sgRNA sequence (~100 bp) using subread-align with default parameters, minFragLength = 30, maxFragLength = 100. For the pooled conjugations, the percentage of mapped sgRNA reads was calculated for each sgRNA relative to the total number of mapped sgRNA reads such that each sgRNA should have ~ 5% of the total mapped sgRNA reads (18 strains for 100% of reads; 1 strain ~ 5%). For the uninduced passaging of CRISPRi pools, the percentage of mapped sgRNA reads was calculated but reported as the L2FC of each guide relative to time zero.

## Data Availability

Gene expression data have been deposited in the GEO Database (GSE313177). All other data are available upon request.

## SUPPLEMENTAL MATERIAL

Supplemental figures mbio.02767-25-s0001.docx Fig. S1-S8.

Supplemental table mbio.02767-25-s0002.xlsx Table S1.

ASM does not own the copyrights to Supplemental Material that may be linked to, or accessed through, an article. The authors have granted ASM a non-exclusive, world-wide license to publish the Supplemental Material files. Please contact the corresponding author directly for reuse.

## References

[1] Baba T, Ara T, Hasegawa M, Takai Y, Okumura Y, Baba M, Datsenko KA, Tomita M, Wanner BL, Mori H. 2006. Construction of Escherichia coli K‐12 in‐frame, single‐gene knockout mutants: the Keio collection. Mol Syst Biol 2:2006.0008. doi:10.1038/msb4100050 PMC1681482 16738554

[2] van Opijnen T, Bodi KL, Camilli A. 2009. Tn-seq: high-throughput parallel sequencing for fitness and genetic interaction studies in microorganisms. Nat Methods 6:767–772. doi:10.1038/nmeth.1377 19767758 PMC2957483

[3] Liberati NT, Urbach JM, Miyata S, Lee DG, Drenkard E, Wu G, Villanueva J, Wei T, Ausubel FM. 2006. An ordered, nonredundant library of Pseudomonas aeruginosa strain PA14 transposon insertion mutants. Proc Natl Acad Sci U S A 103:2833–2838. doi:10.1073/pnas.0511100103 16477005 PMC1413827

[4] McGinness KE, Baker TA, Sauer RT. 2006. Engineering controllable protein degradation. Mol Cell 22:701–707. doi:10.1016/j.molcel.2006.04.027 16762842

[5] Kim J-H, Wei J-R, Wallach JB, Robbins RS, Rubin EJ, Schnappinger D. 2011. Protein inactivation in mycobacteria by controlled proteolysis and its application to deplete the beta subunit of RNA polymerase. Nucleic Acids Res 39:2210–2220. doi:10.1093/nar/gkq1149 21075796 PMC3064785

[6] Carrillo Rincón AF, Farny NG. 2023. Unlocking the strength of inducible promoters in Gram-negative bacteria. Microb Biotechnol 16:961–976. doi:10.1111/1751-7915.14219 36738130 PMC10128130

[7] Qi LS, Larson MH, Gilbert LA, Doudna JA, Weissman JS, Arkin AP, Lim WA. 2013. Repurposing CRISPR as an RNA-guided platform for sequence-specific control of gene expression. Cell 152:1173–1183. doi:10.1016/j.cell.2013.02.022 23452860 PMC3664290

[8] Todor H, Silvis MR, Osadnik H, Gross CA. 2021. Bacterial CRISPR screens for gene function. Curr Opin Microbiol 59:102–109. doi:10.1016/j.mib.2020.11.005 33285498 PMC8331264

[9] Depardieu F, Bikard D. 2020. Gene silencing with CRISPRi in bacteria and optimization of dCas9 expression levels. Methods 172:61–75. doi:10.1016/j.ymeth.2019.07.024 31377338

[10] Larson MH, Gilbert LA, Wang X, Lim WA, Weissman JS, Qi LS. 2013. CRISPR interference (CRISPRi) for sequence-specific control of gene expression. Nat Protoc 8:2180–2196. doi:10.1038/nprot.2013.132 24136345 PMC3922765

[11] Peters JM, Colavin A, Shi H, Czarny TL, Larson MH, Wong S, Hawkins JS, Lu CHS, Koo B-M, Marta E, Shiver AL, Whitehead EH, Weissman JS, Brown ED, Qi LS, Huang KC, Gross CA. 2016. A comprehensive, CRISPR-based functional analysis of essential genes in bacteria. Cell 165:1493–1506. doi:10.1016/j.cell.2016.05.003 27238023 PMC4894308

[12] Liu X, Gallay C, Kjos M, Domenech A, Slager J, van Kessel SP, Knoops K, Sorg RA, Zhang J-R, Veening J-W. 2017. High-throughput CRISPRi phenotyping identifies new essential genes in Streptococcus pneumoniae. Mol Syst Biol 13:931. doi:10.15252/msb.20167449 28490437 PMC5448163

[13] Peters JM, Koo B-M, Patino R, Heussler GE, Hearne CC, Qu J, Inclan YF, Hawkins JS, Lu CHS, Silvis MR, Harden MM, Osadnik H, Peters JE, Engel JN, Dutton RJ, Grossman AD, Gross CA, Rosenberg OS. 2019. Enabling genetic analysis of diverse bacteria with mobile-CRISPRi. Nat Microbiol 4:244–250. doi:10.1038/s41564-018-0327-z 30617347 PMC6424567

[14] Zhang Y, Zhang T, Xiao X, Wang Y, Kawalek A, Ou J, Ren A, Sun W, de Bakker V, Liu Y, Li Y, Yang L, Ye L, Jia N, Veening J-W, Liu X. 2025. CRISPRi screen identifies FprB as a synergistic target for gallium therapy in Pseudomonas aeruginosa. Nat Commun 16:5870. doi:10.1038/s41467-025-61208-z 40595632 PMC12216716

[15] Tan SZ, Reisch CR, Prather KLJ. 2018. A robust CRISPR interference gene repression system in Pseudomonas. J Bacteriol 200:e00575-17. doi:10.1128/JB.00575-17 29311279 PMC5847647

[16] Rock JM, Hopkins FF, Chavez A, Diallo M, Chase MR, Gerrick ER, Pritchard JR, Church GM, Rubin EJ, Sassetti CM, Schnappinger D, Fortune SM. 2017. Programmable transcriptional repression in mycobacteria using an orthogonal CRISPR interference platform. Nat Microbiol 2:16274. doi:10.1038/nmicrobiol.2016.274 28165460 PMC5302332

[17] Bosch B, DeJesus MA, Poulton NC, Zhang W, Engelhart CA, Zaveri A, Lavalette S, Ruecker N, Trujillo C, Wallach JB, Li S, Ehrt S, Chait BT, Schnappinger D, Rock JM. 2021. Genome-wide gene expression tuning reveals diverse vulnerabilities of M. tuberculosis. Cell 184:4579–4592. doi:10.1016/j.cell.2021.06.033 34297925 PMC8382161

[18] Tacconelli E, Carrara E, Savoldi A, Harbarth S, Mendelson M, Monnet DL, Pulcini C, Kahlmeter G, Kluytmans J, Carmeli Y, et al.. 2018. Discovery, research, and development of new antibiotics: the WHO priority list of antibiotic-resistant bacteria and tuberculosis. Lancet Infect Dis 18:318–327. doi:10.1016/S1473-3099(17)30753-3 29276051

[19] Meisner J, Goldberg JB. 2016. The Escherichia coli rhaSR-PrhaBAD inducible promoter system allows tightly controlled gene expression over a wide range in Pseudomonas aeruginosa. Appl Environ Microbiol 82:6715–6727. doi:10.1128/AEM.02041-16 27613678 PMC5086565

[20] Noirot-Gros M-F, Forrester S, Malato G, Larsen PE, Noirot P. 2019. CRISPR interference to interrogate genes that control biofilm formation in Pseudomonas fluorescens. Sci Rep 9:15954. doi:10.1038/s41598-019-52400-5 31685917 PMC6828691

[21] Xiang L, Qi F, Jiang L, Tan J, Deng C, Wei Z, Jin S, Huang G. 2020. CRISPR-dCas9-mediated knockdown of prtR, an essential gene in Pseudomonas aeruginosa. Lett Appl Microbiol 71:386–393. doi:10.1111/lam.13337 32506497

[22] Li XZ, Livermore DM, Nikaido H. 1994. Role of efflux pump(s) in intrinsic resistance of Pseudomonas aeruginosa: resistance to tetracycline, chloramphenicol, and norfloxacin. Antimicrob Agents Chemother 38:1732–1741. doi:10.1128/AAC.38.8.1732 7986003 PMC284630

[23] Ozer B, Duran N, Onlen Y, Savas L. 2012. Efflux pump genes and antimicrobial resistance of Pseudomonas aeruginosa strains isolated from lower respiratory tract infections acquired in an intensive care unit. J Antibiot (Tokyo) 65:9–13. doi:10.1038/ja.2011.102 22086166

[24] Choi K-H, Schweizer HP. 2006. Mini-Tn7 insertion in bacteria with single attTn7 sites: example Pseudomonas aeruginosa. Nat Protoc 1:153–161. doi:10.1038/nprot.2006.24 17406227

[25] Wong AI, Rock JM. 2021. CRISPR interference (CRISPRi) for targeted gene silencing in mycobacteria. Methods Mol Biol 2314:343–364. doi:10.1007/978-1-0716-1460-0_16 34235662

[26] Jinek M, Chylinski K, Fonfara I, Hauer M, Doudna JA, Charpentier E. 2012. A programmable dual-RNA–guided DNA endonuclease in adaptive bacterial immunity. Science 337:816–821. doi:10.1126/science.1225829 22745249 PMC6286148

[27] de Jong NWM, van der Horst T, van Strijp JAG, Nijland R. 2017. Fluorescent reporters for markerless genomic integration in Staphylococcus aureus. Sci Rep 7:43889. doi:10.1038/srep43889 28266573 PMC5339689

[28] Shcherbo D, Murphy CS, Ermakova GV, Solovieva EA, Chepurnykh TV, Shcheglov AS, Verkhusha VV, Pletnev VZ, Hazelwood KL, Roche PM, Lukyanov S, Zaraisky AG, Davidson MW, Chudakov DM. 2009. Far-red fluorescent tags for protein imaging in living tissues. Biochem J 418:567–574. doi:10.1042/BJ20081949 19143658 PMC2893397

[29] Bikard D, Jiang W, Samai P, Hochschild A, Zhang F, Marraffini LA. 2013. Programmable repression and activation of bacterial gene expression using an engineered CRISPR-Cas system. Nucleic Acids Res 41:7429–7437. doi:10.1093/nar/gkt520 23761437 PMC3753641

[30] McMackin EAW, Corley JM, Karash S, Marden J, Wolfgang MC, Yahr TL. 2021. Cautionary notes on the use of arabinose- and rhamnose-inducible expression vectors in Pseudomonas aeruginosa. J Bacteriol 203:e00224-21. doi:10.1128/JB.00224-21 34096777 PMC8297530

[31] Poulsen BE, Yang R, Clatworthy AE, White T, Osmulski SJ, Li L, Penaranda C, Lander ES, Shoresh N, Hung DT. 2019. Defining the core essential genome of Pseudomonas aeruginosa. Proc Natl Acad Sci U S A 116:10072–10080. doi:10.1073/pnas.1900570116 31036669 PMC6525520

[32] Ward RD, Tran JS, Banta AB, Bacon EE, Rose WE, Peters JM. 2024. Essential gene knockdowns reveal genetic vulnerabilities and antibiotic sensitivities in Acinetobacter baumannii. mBio 15:e02051-23. doi:10.1128/mbio.02051-23 PMC10865783 38126769

[33] Poulsen BE, Warrier T, Barkho S, Bagnall J, Romano KP, White T, Yu X, Kawate T, Nguyen PH, Raines K, Ferrara K, Golas AL, FitzGerald M, Boeszoermenyi A, Kaushik V, Serrano-Wu M, Shoresh N, Hung DT. 2025. Discovery of a Pseudomonas aeruginosa-specific small molecule targeting outer membrane protein OprH-LPS interaction by a multiplexed screen. Cell Chem Biol 32:307–324. doi:10.1016/j.chembiol.2024.12.001 39732052 PMC12117999

[34] Cacace E, Kritikos G, Typas A. 2017. Chemical genetics in drug discovery. Curr Opin Syst Biol 4:35–42. doi:10.1016/j.coisb.2017.05.020 32715163 PMC7371212

[35] Simpkins SW, Nelson J, Deshpande R, Li SC, Piotrowski JS, Wilson EH, Gebre AA, Safizadeh H, Okamoto R, Yoshimura M, Costanzo M, Yashiroda Y, Ohya Y, Osada H, Yoshida M, Boone C, Myers CL. 2018. Predicting bioprocess targets of chemical compounds through integration of chemical-genetic and genetic interactions. PLoS Comput Biol 14:e1006532. doi:10.1371/journal.pcbi.1006532 30376562 PMC6226211

[36] Wildenhain J, Spitzer M, Dolma S, Jarvik N, White R, Roy M, Griffiths E, Bellows DS, Wright GD, Tyers M. 2016. Systematic chemical-genetic and chemical-chemical interaction datasets for prediction of compound synergism. Sci Data 3:160095. doi:10.1038/sdata.2016.95 27874849 PMC5127411

[37] Choudhery S, DeJesus MA, Srinivasan A, Rock J, Schnappinger D, Ioerger TR. 2024. A dose-response model for statistical analysis of chemical genetic interactions in CRISPRi screens. PLoS Comput Biol 20:e1011408. doi:10.1371/journal.pcbi.1011408 38768228 PMC11104602

[38] Collins JA, Osheroff N. 2024. Gyrase and topoisomerase IV: recycling old targets for new antibacterials to combat fluoroquinolone resistance. ACS Infect Dis 10:1097–1115. doi:10.1021/acsinfecdis.4c00128 38564341 PMC11019561

[39] Kohanski MA, Dwyer DJ, Wierzbowski J, Cottarel G, Collins JJ. 2008. Mistranslation of membrane proteins and two-component system activation trigger antibiotic-mediated cell death. Cell 135:679–690. doi:10.1016/j.cell.2008.09.038 19013277 PMC2684502

[40] Romano KP, Bagnall J, Warrier T, Sullivan J, Ferrara K, Orzechowski M, Nguyen PH, Raines K, Livny J, Shoresh N, Hung DT. 2024. Perturbation-specific transcriptional mapping for unbiased target elucidation of antibiotics. Proc Natl Acad Sci USA 121:e2409747121. doi:10.1073/pnas.2409747121 39467118 PMC11551328

[41] Palmer AC, Kishony R. 2014. Opposing effects of target overexpression reveal drug mechanisms. Nat Commun 5:4296. doi:10.1038/ncomms5296 24980690 PMC4408919

[42] Sperandeo P, Cescutti R, Villa R, Di Benedetto C, Candia D, Dehò G, Polissi A. 2007. Characterization of lptA and lptB, two essential genes implicated in lipopolysaccharide transport to the outer membrane of Escherichia coli. J Bacteriol 189:244–253. doi:10.1128/JB.01126-06 17056748 PMC1797204

[43] Tran AX, Trent MS, Whitfield C. 2008. The LptA protein of Escherichia coli is a periplasmic lipid A-binding protein involved in the lipopolysaccharide export pathway. J Biol Chem 283:20342–20349. doi:10.1074/jbc.M802503200 18480051 PMC2459282

[44] Keren L, Hausser J, Lotan-Pompan M, Vainberg Slutskin I, Alisar H, Kaminski S, Weinberger A, Alon U, Milo R, Segal E. 2016. Massively parallel interrogation of the effects of gene expression levels on fitness. Cell 166:1282–1294. doi:10.1016/j.cell.2016.07.024 27545349

[45] Jost M, Santos DA, Saunders RA, Horlbeck MA, Hawkins JS, Scaria SM, Norman TM, Hussmann JA, Liem CR, Gross CA, Weissman JS. 2020. Titrating gene expression using libraries of systematically attenuated CRISPR guide RNAs. Nat Biotechnol 38:355–364. doi:10.1038/s41587-019-0387-5 31932729 PMC7065968

[46] Fivenson EM, Bernhardt TG. 2020. An essential membrane protein modulates the proteolysis of LpxC to control lipopolysaccharide synthesis in Escherichia coli. mBio 11:e00939-20. doi:10.1128/mBio.00939-20 32430473 PMC7240159

[47] Pahil KS, Gilman MSA, Baidin V, Clairfeuille T, Mattei P, Bieniossek C, Dey F, Muri D, Baettig R, Lobritz M, Bradley K, Kruse AC, Kahne D. 2024. A new antibiotic traps lipopolysaccharide in its intermembrane transporter. Nature 625:572–577. doi:10.1038/s41586-023-06799-7 38172635 PMC10794137

[48] Lee J, Xue M, Wzorek JS, Wu T, Grabowicz M, Gronenberg LS, Sutterlin HA, Davis RM, Ruiz N, Silhavy TJ, Kahne DE. 2016. Characterization of a stalled complex on the β-barrel assembly machine. Proc Natl Acad Sci USA 113:8717–8722. doi:10.1073/pnas.1604100113 27439868 PMC4978274

[49] Miranda SW, Asfahl KL, Dandekar AA, Greenberg EP. 2022. Pseudomonas aeruginosa quorum sensing. Adv Exp Med Biol 1386:95–115. doi:10.1007/978-3-031-08491-1_4 36258070 PMC9942581

[50] Cui L, Vigouroux A, Rousset F, Varet H, Khanna V, Bikard D. 2018. A CRISPRi screen in E. coli reveals sequence-specific toxicity of dCas9. Nat Commun 9:1912. doi:10.1038/s41467-018-04209-5 29765036 PMC5954155

[51] Wang T, Guan C, Guo J, Liu B, Wu Y, Xie Z, Zhang C, Xing X-H. 2018. Pooled CRISPR interference screening enables genome-scale functional genomics study in bacteria with superior performance. Nat Commun 9:2475. doi:10.1038/s41467-018-04899-x 29946130 PMC6018678

[52] Rousset F, Cui L, Siouve E, Becavin C, Depardieu F, Bikard D. 2018. Genome-wide CRISPR-dCas9 screens in E. coli identify essential genes and phage host factors. PLoS Genet 14:e1007749. doi:10.1371/journal.pgen.1007749 30403660 PMC6242692

[53] Camsund D, Lawson MJ, Larsson J, Jones D, Zikrin S, Fange D, Elf J. 2020. Time-resolved imaging-based CRISPRi screening. Nat Methods 17:86–92. doi:10.1038/s41592-019-0629-y 31740817

[54] Mutalik VK, Adler BA, Rishi HS, Piya D, Zhong C, Koskella B, Kutter EM, Calendar R, Novichkov PS, Price MN, Deutschbauer AM, Arkin AP. 2020. High-throughput mapping of the phage resistance landscape in E. coli. PLoS Biol 18:e3000877. doi:10.1371/journal.pbio.3000877 33048924 PMC7553319

[55] Hawkins JS, Silvis MR, Koo B-M, Peters JM, Osadnik H, Jost M, Hearne CC, Weissman JS, Todor H, Gross CA. 2020. Mismatch-CRISPRi reveals the co-varying expression-fitness relationships of essential genes in Escherichia coli and Bacillus subtilis. Cell Syst 11:523–535. doi:10.1016/j.cels.2020.09.009 33080209 PMC7704046

[56] de Wet TJ, Winkler KR, Mhlanga M, Mizrahi V, Warner DF. 2020. Arrayed CRISPRi and quantitative imaging describe the morphotypic landscape of essential mycobacterial genes. eLife 9:1–36. doi:10.7554/eLife.60083 PMC7647400 33155979

[57] Romano KP, Warrier T, Poulsen BE, Nguyen PH, Loftis AR, Saebi A, Pentelute BL, Hung DT. 2019. Mutations in pmrB confer cross-resistance between the LptD inhibitor POL7080 and colistin in Pseudomonas aeruginosa. Antimicrob Agents Chemother 63:e00511-19. doi:10.1128/AAC.00511-19 31235628 PMC6709506

[58] Hoang TT, Kutchma AJ, Becher A, Schweizer HP. 2000. Integration-proficient plasmids for Pseudomonas aeruginosa: site-specific integration and use for engineering of reporter and expression strains. Plasmid 43:59–72. doi:10.1006/plas.1999.1441 10610820

[59] Shishkin AA, Giannoukos G, Kucukural A, Ciulla D, Busby M, Surka C, Chen J, Bhattacharyya RP, Rudy RF, Patel MM, Novod N, Hung DT, Gnirke A, Garber M, Guttman M, Livny J. 2015. Simultaneous generation of many RNA-seq libraries in a single reaction. Nat Methods 12:323–325. doi:10.1038/nmeth.3313 25730492 PMC4712044

[60] Zhu YY, Machleder EM, Chenchik A, Li R, Siebert PD. 2001. Reverse transcriptase template switching: a SMART. BioTechniques 30:892–897. doi:10.2144/01304pf02 11314272

[61] Love MI, Huber W, Anders S. 2014. Moderated estimation of fold change and dispersion for RNA-seq data with DESeq2. Genome Biol 15:550. doi:10.1186/s13059-014-0550-8 25516281 PMC4302049

[62] Szklarczyk D, Kirsch R, Koutrouli M, Nastou K, Mehryary F, Hachilif R, Gable AL, Fang T, Doncheva NT, Pyysalo S, Bork P, Jensen LJ, von Mering C. 2023. The STRING database in 2023: protein-protein association networks and functional enrichment analyses for any sequenced genome of interest. Nucleic Acids Res 51:D638–D646. doi:10.1093/nar/gkac1000 36370105 PMC9825434

[63] The Broad Institute. 2016. Morpheus. https://software.broadinstitute.org/morpheus.

[64] Bolger AM, Lohse M, Usadel B. 2014. Trimmomatic: a flexible trimmer for Illumina sequence data. Bioinformatics 30:2114–2120. doi:10.1093/bioinformatics/btu170 24695404 PMC4103590

[65] Liao Y, Smyth GK, Shi W. 2013. The Subread aligner: fast, accurate and scalable read mapping by seed-and-vote. Nucleic Acids Res 41:e108. doi:10.1093/nar/gkt214 23558742 PMC3664803

