# Supplementary material for: An inducible CRISPRi system for phenotypic analysis of essential genes in Pseudomonas aeruginosa
Source: mBio. 2026 Feb 11;17(3):e02767-25. doi: 10.1128/mbio.02767-25 (PMC12977507; doi:10.1128/mbio.02767-25)
Supplement: Supplemental figures — Fig. S1-S8. [file mbio.02767-25-s0001.docx]

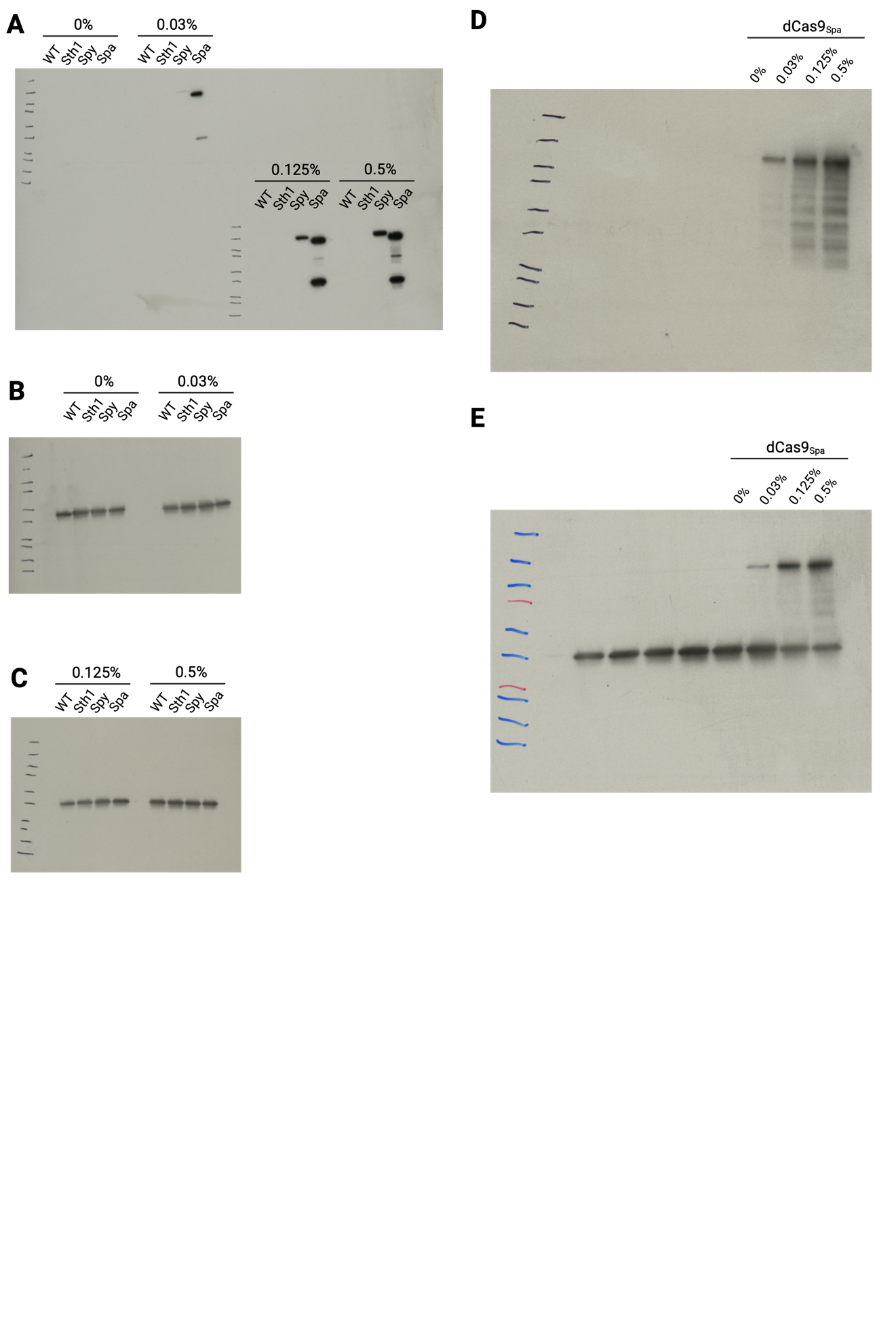


Figure S1. Expression tests of dCas9 variants in *P. aeruginosa*. (A) Western blot probing for *S. thermophilus* (Sth1), *S. pyogenes* (Spy), or *S. pasteurianus* (Spa) dCas9 expression in 0%, 0.03%, 0.125%, or 0.5% arabinose. (B and C) Western blot probing for loading control protein RpoA in A. (D) Western blot probing for dCas9_Spy_ expression in 0%, 0.03%, 0.125%, and 0.5% arabinose with a dCas9_Spy_-specific antibody after induction. (E) Western blot probing for loading control protein RpoA in D.


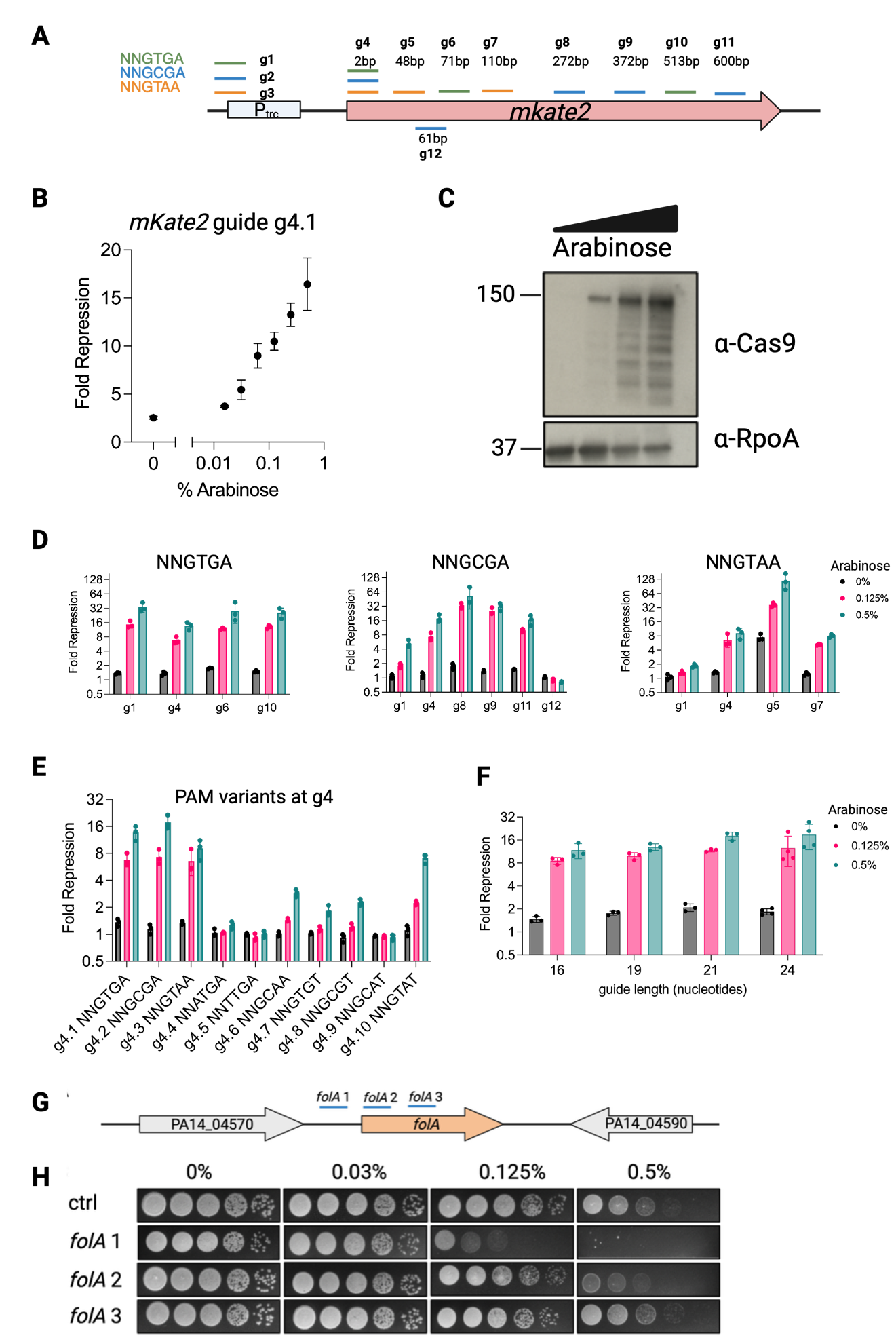


Figure S2. Characterization of the arabinose-based CRISPRi^A^ system. (A) Schematic of the open reading frame of the fluorescent reporter mKate2 with PAMs and sgRNA locations highlighted. Base pairs indicate distance from *mKate2* start codon. (B) mKate2 fluorescence repression (normalized to control sgRNA) at varying arabinose doses using sgRNA g4.1 that targeted the start codon of *mKate2*. Fluorescence reduction showed direct correlation with arabinose concentration and thus dCas9_Spa_ expression levels. (C) Western blot probing for dCas9_Spy_ expression in 0%, 0.03%, 0.125%, and 0.5% arabinose with a dCas9-specific antibody after induction indicated protein degradation at higher arabinose doses. (D) Effect of varying guide location with the three reported dCas9_Spa_-specific PAM sequences on mKate2 fluorescence repression. The guides bound to the promoter or open reading frame of *mKate2* as indicated in panel A. NNGTGA and NNGCGA were equally effective irrespective of guide location while NNGTAA was weaker and displayed location bias. (E) Impact of PAM sequence engineered in the start codon-targeting sgRNA g4 on dCas9_Spa_ activity as measured by mKate2 fluorescence repression. NNGTGA and NNGCGA led to the highest mKate2 depletion (16-fold fluorescence repression) and were closely followed by NNGTAA and the newly designed NNGTAT (~8-fold repression). (F) Impact of length of the targeting region of the guide. Targeting region length between 16 and 24 nucleotides (nt) showed similar extent of fluorescence repression. (G) Schematic of guide design to target *folA* using CRISPRi^A^. sgRNAs were designed to target the promoter region (*folA1*) or the coding sequence (*folA2*, *folA3*). (H) Growth defects from *folA* knockdown. Spot dilutions of CRISPRi^A^ strains with control or *folA* sgRNAs grown on LB agar with varying arabinose concentrations. Growth defects were more apparent when the promoter region was targeted.


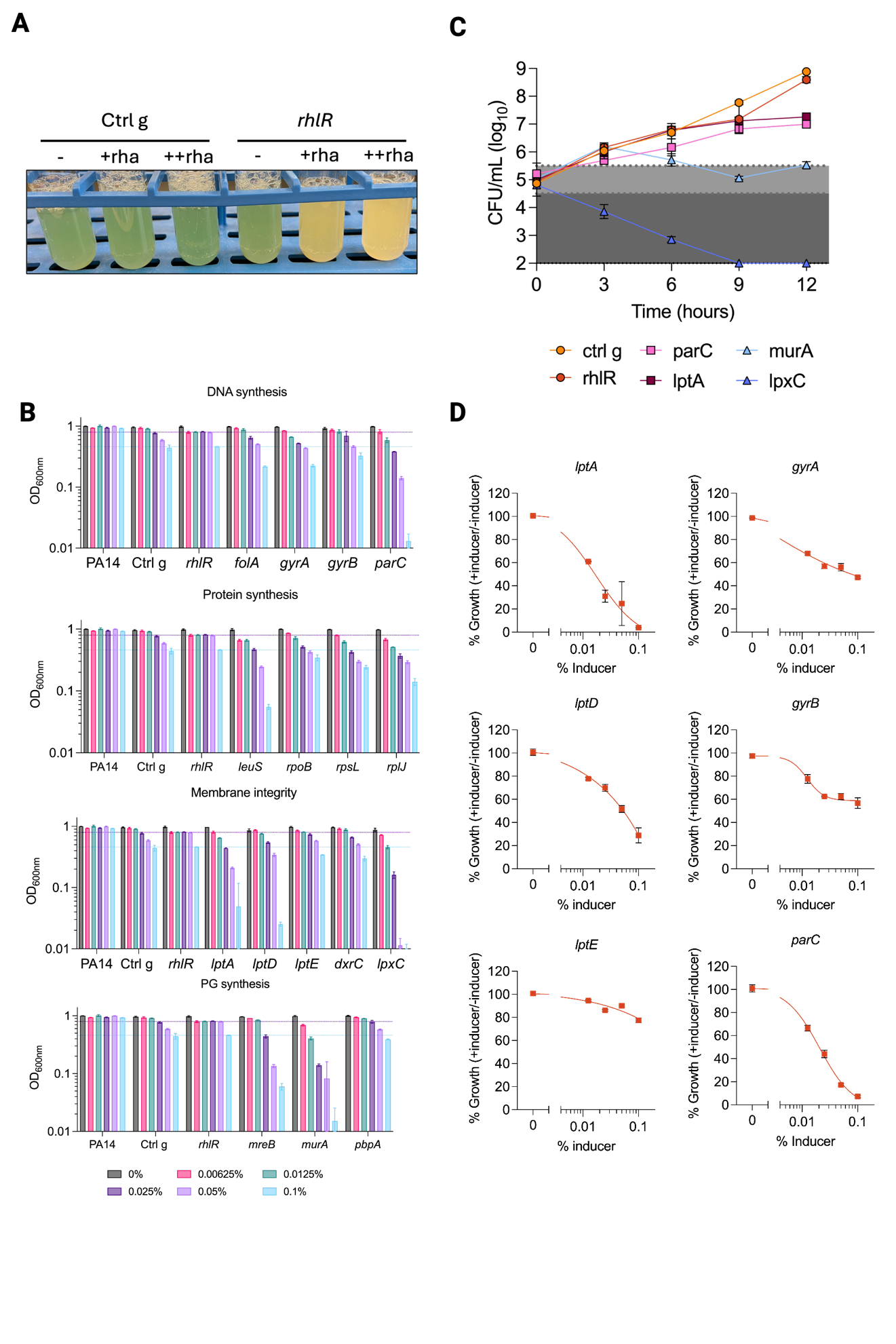


Figure S3. Phenotypic effects of CRISPRi^R^ knockdown. (A) CRISPRi^R^ was effective against endogenous non-essential gene, *rhlR*, but had minimal impact on growth. Knockdown with 0.0125% rhamnose (+) or 0.05% rhamnose (++) exposure overnight caused significant reduction of pigmented pyocyanin, whose production is regulated by RhlR, relative to the *control*-CRISPRi strain. (B) CRISPRi^R^ targeting of endogenous essential genes led to rhamnose dose-dependent growth inhibition. Individual CRISPRi strains were grown in LB broth in the presence of 0%, 0.00625%, 0.0125%, 0.025%, 0.05% or 0.01% rhamnose and optical density at 600nm (OD_600nm_) was measured after 24 hours. Dashed lines indicate OD_600nm_ after knockdown of the non-essential gene *rhlR*. (C) Kill kinetics of CRISPRi^R^ strains grown in LB with 0.05% rhamnose. Effect of knockdown on growth is shown with background colour (white – slowed growth, light grey – bacteriostatic, dark grey – bactericidal). (D) Dose-response curves of the growth of CRISPRi^R^ strains targeting PA14 genes involved in LPS transport or related to DNA synthesis at varying concentrations of the inducer, rhamnose. CRISPRi^R^ strains were grown in LB broth with varying concentrations of rhamnose for 24 hours and OD_600nm_ was read to measure growth. It was normalized to uninduced conditions and plotted as %Growth. The degree of growth defect is gene-specific and pathway-independent.


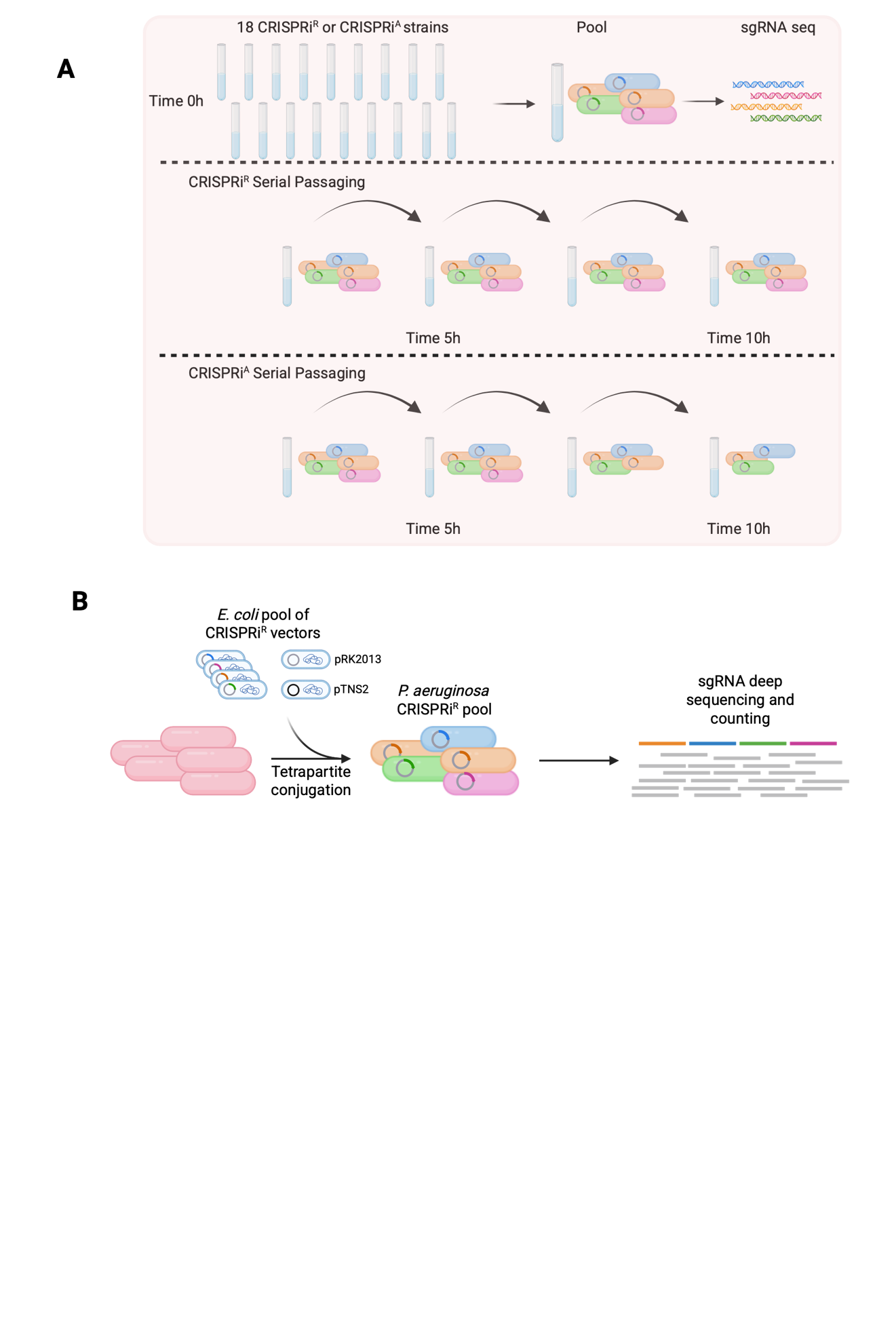


Figure S4. CRISPRi^R^ facilitates pooled CRISPRi library construction in *P. aeruginosa*. (A) Schematic showing the propagation of CRISPRi pools without inducer. All CRISPRi strains were grown arrayed and then pooled at equal cell densities. Pooled strains were grown from OD_600_ 0.025 until mid-log and passaged back to OD_600_ 0.2 for 5 cycles. Aliquots were collected after 0, 5, and 10 hours of passaging, gDNA extracted and processed for sgRNA-targeted amplicon sequencing. (B) Schematic showing the pooled CRISPRi conjugation strategy. The tetrapartite mating is set up between recipient PA14::*dcas9*, *E. coli* helper pRK2013, *E. coli* helper pTNS2, and a pool of *E. coli* donor CRISPRi^R^ strains (17 different *E. coli* CRISPRi^R^ sgRNA strains pooled at equal cell density). After selection of true PA14 CRISPRi^R^ conjugants with irgasan (5 µg/mL) and gentamicin (15 µg/mL), colonies were pooled together, gDNA extracted and processed for sgRNA-targeted amplicon sequencing.


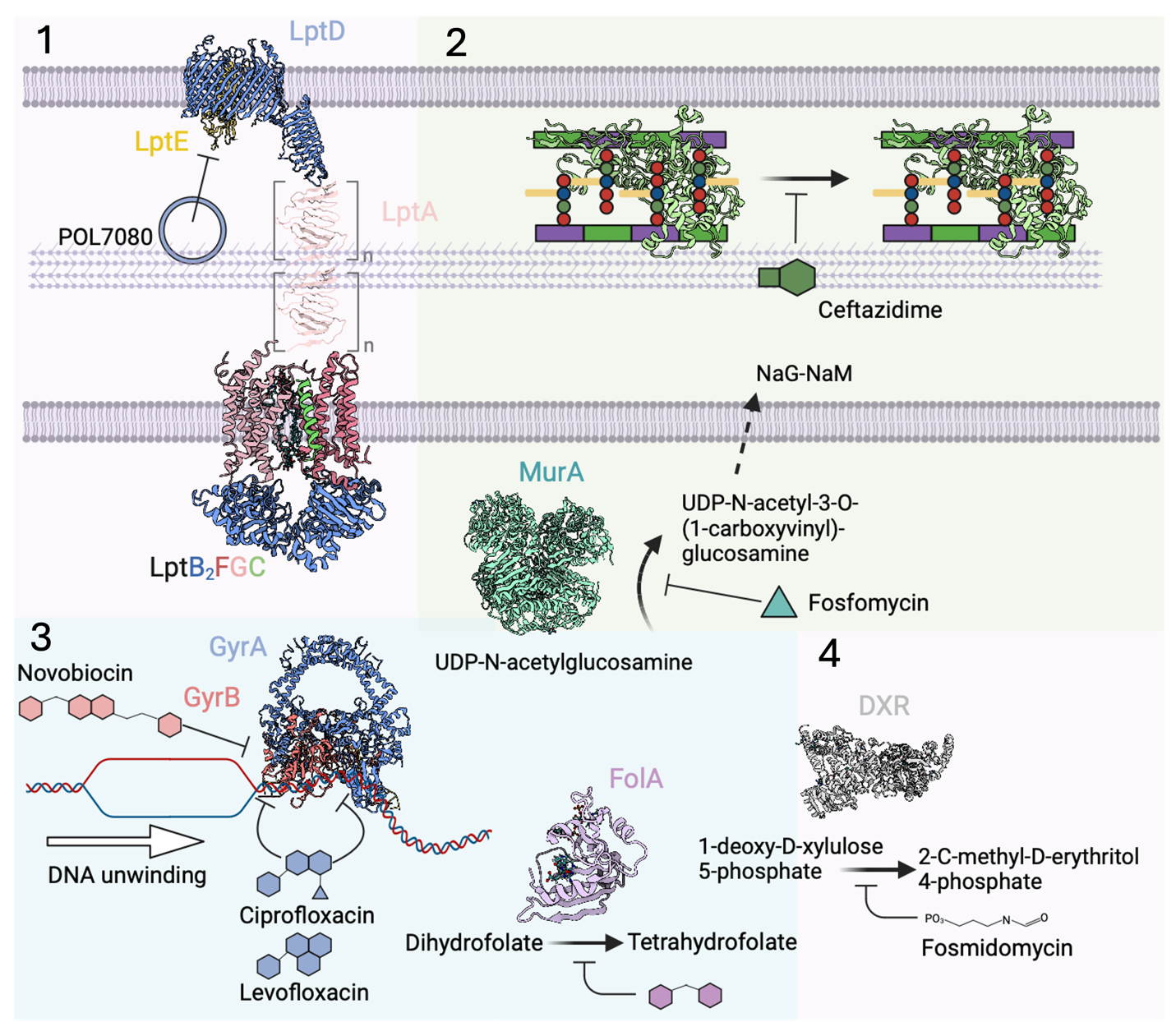


Figure S5. Cartoon of chemical inhibitors and their targets in (1) LPS trafficking, (2) peptidoglycan synthesis, (3) DNA synthesis-related, and (4) dxr pathways in *P. aeruginosa*.


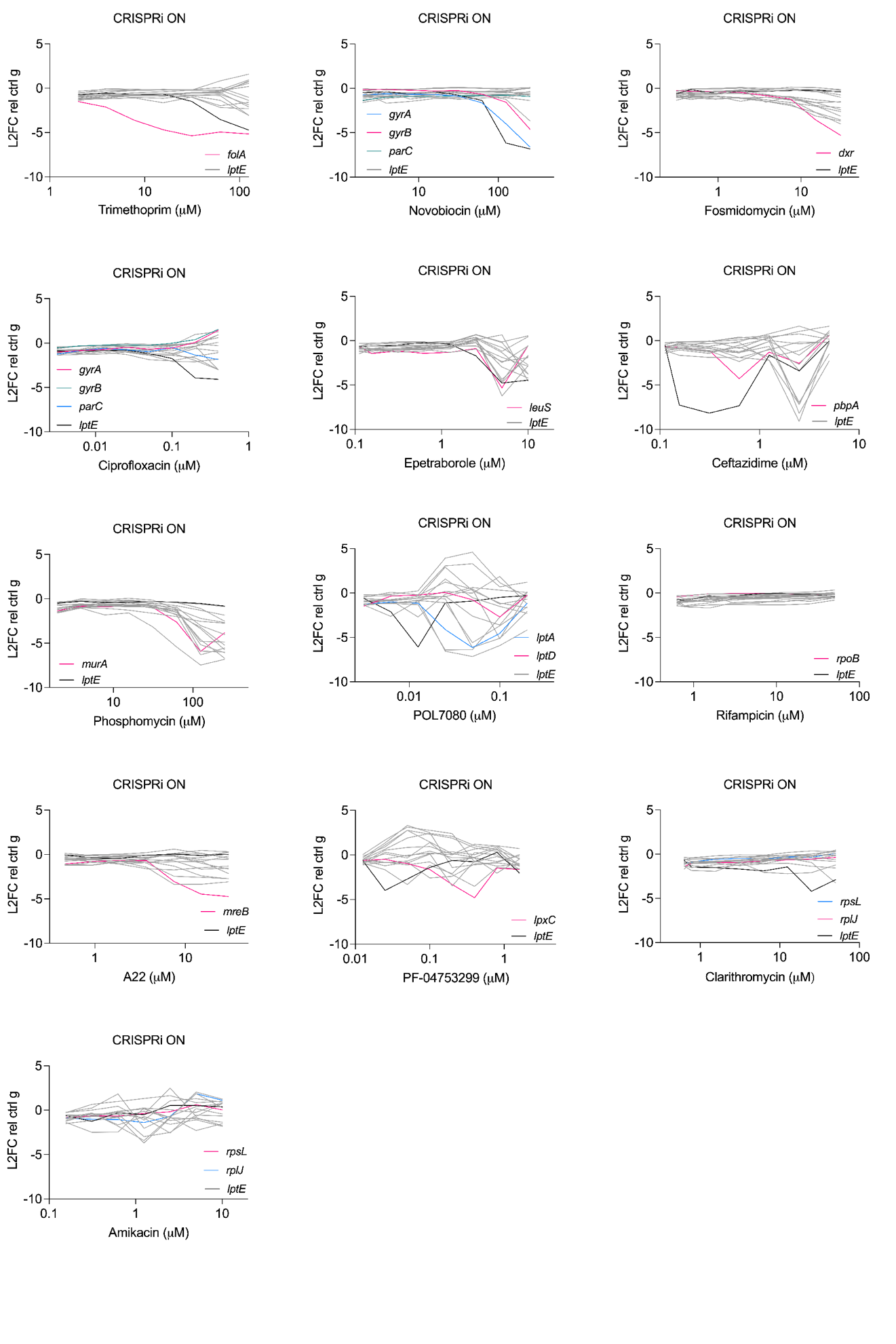


Figure S6. Dose-response curves of the log_2_ fold change (L2FC) in growth of CRISPRi^R^ strains relative to *control*-CRISPRi^R^ strain. CRISPRi^R^ strains were grown in sub-lethal rhamnose doses. *folA-*, *gyrA-*, *gyrB-*, *lptE-*, *rpoB-*, and *pbpA*-CRISPRi strains were grown in 0.05% rhamnose. *parC*-, *lptA-*, *lptD-*, *dxr-*, *mreB-*, *leuS-*, *rpsL*, and *rplJ*-CRISPRi strains were grown in 0.025% rhamnose. *lpxC-* and *murA*-CRISPRi strains were grown in 0.0125% rhamnose. Growth was measured with optical density at 600 nm after 24 hours in the presence of chemical inhibitor. Fold change in growth relative to *control*-CRISPRi strain grown with identical rhamnose doses is plotted. The putative genetic target of the chemical inhibitor is highlighted in pink. *gyrB* and *parC* are highlighted as non-binding targets and alternative targets, respectively for ciprofloxacin. *lptA* and *lptE* are highlighted as on pathway targets for POL7080. *rplJ* and *rpsL* are highlighted as on pathway targets for amikacin and clarithromycin. *lptE*-CRISPRi strain is broadly sensitized to many chemical inhibitors and is highlighted for all chemical inhibitors.


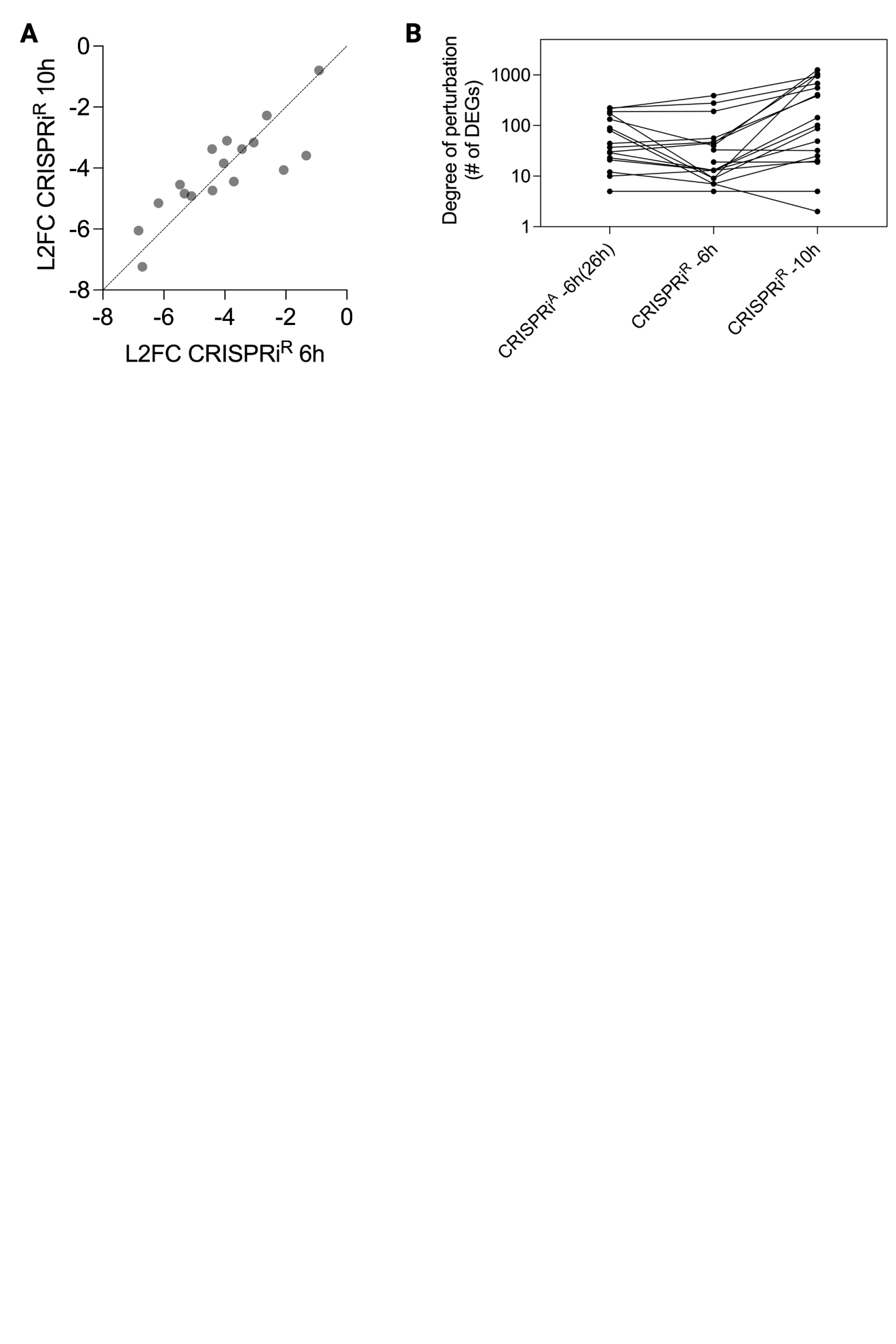


Figure S7. Tighter control and extended CRISPRi^R^ activation results in greater transcriptomic perturbation. (A) CRISPRi^R^ knockdown analyzed by RNA-seq and quantified using log_2_ fold change (L2FC) of target gene relative to *control*-CRISPRi strain after 6 and 10 hours of growth in 0.05% rhamnose. Each point is an individual CRISPRi^R^ strain. Measuring L2FC at 10 hours required a modified experimental set up: strains were grown in LB 0.05% rhamnose from OD_600nm_ 0.05 to 0.5 (first 6 hours) and back diluted to OD_600nm_ 0.1 and allowed to grow until OD_600nm_ 0.3 (an additional 4 hours). Back dilution of the CRISPRi^R^ strains prevented entry into stationary phase for accurate transcriptional profiling at the 10-hour time point. (B) Differentially expressed genes (DEGs) from target gene knockdown using CRISPRi^A^ after 6 hours of induction with 0.5% arabinose (~effectively 26 hours of induction due to the leaky system) or CRISPRi^R^ after 6 and 10 hours of induction with 0.05% rhamnose. The extended induction of 10 hours with CRISPRi^R^ captured a more comprehensive transcriptional perturbation downstream of target depletion for most genes as reflected in the increase in DEGs relative to CRISPRi^A^ or the shorter 6-hour induction with CRISPRi^R^.


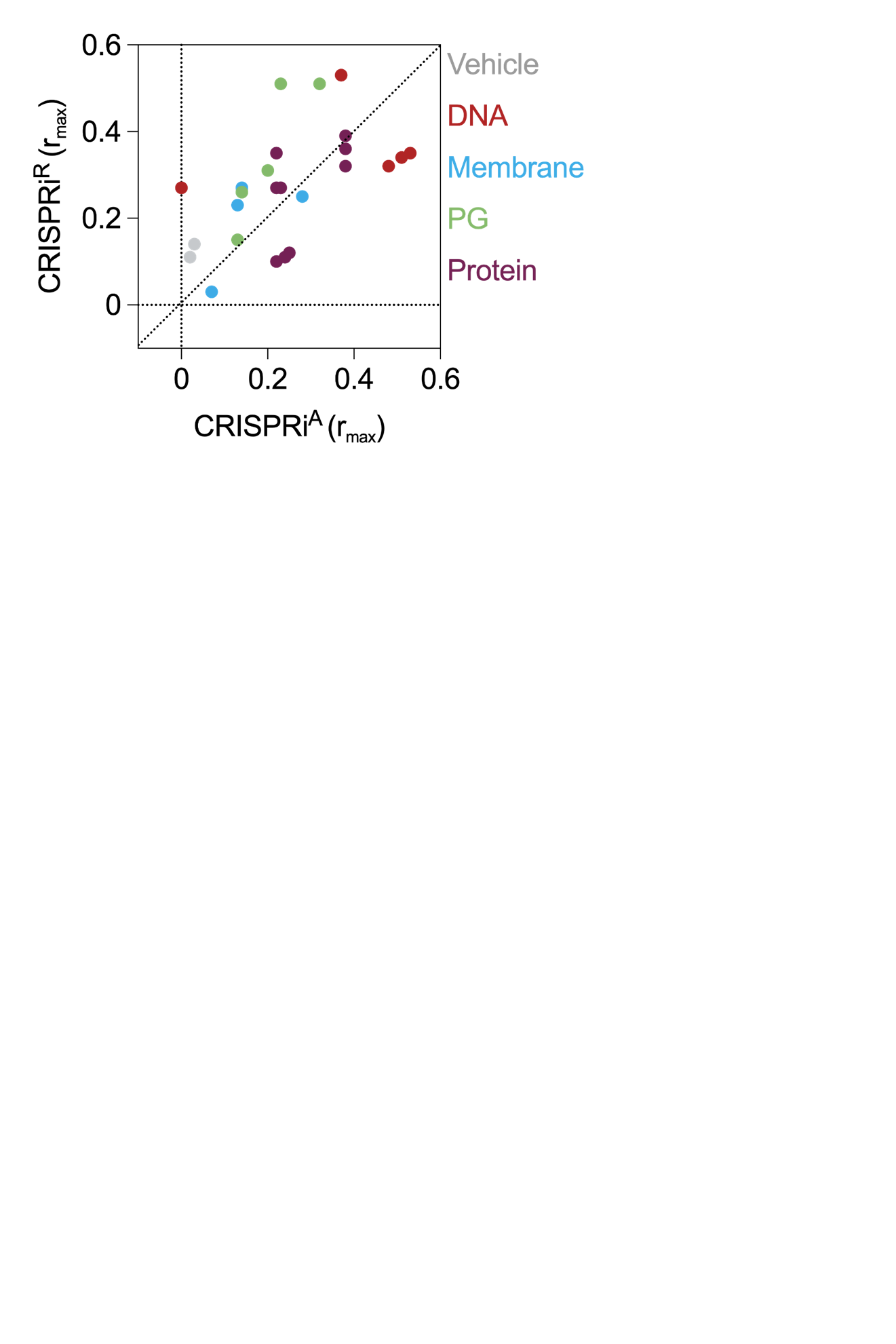


Figure S8. Pearson correlations from PerSpecTM analysis of chemical inhibitors queried against CRISPRi strains are higher from CRISPRi^R^ target knockdown. Maximum Pearson correlations (r_max_) for each chemical inhibitor and its known target knockdown using each CRISPRi system are shown and color coordinated by mechanism of action (Vehicle: water, DMSO; DNA: ciprofloxacin, levofloxacin, cisplatin, novobiocin, trimethoprim; Membrane: fosmidomycin, PF3299, POL7080, colistin; PG: A22, phosphomycin, carbenicillin, imipenem, ceftazidime; Protein: rifampicin, amikacin, gentamicin, tobramycin, tetracycline, doxycycline, minocycline, clarithromycin, erythromycin). CRISPRi^R^ r_max_ values are from the heatmap in Figure 5A; CRISPRi^A^ r_max_ values are from Romano, K. P. *et al.* Perturbation-specific transcriptional mapping for unbiased target elucidation of antibiotics. *Proc Natl Acad Sci U S A* **121**, (2024).
